# An epigenetic perspective on neonatal encephalopathy with suspected hypoxic ischaemic encephalopathy

**DOI:** 10.1186/s13148-025-01984-z

**Published:** 2025-12-08

**Authors:** Priyal Mistry, Juanita Mellet, Chrisna Durandt, Izelle Smuts, Michael S. Pepper

**Affiliations:** 1 Institute for Cellular and Molecular Medicine, Department of Immunology, SAMRC Extramural Unit for Stem Cell Research and Therapy, University of Pretoria, Pretoria, 0084 South Africa; 2 Department of Paediatrics and Child Health, Steve Biko Academic Hospital, University of Pretoria, Pretoria, 0084 South Africa

**Keywords:** Neonatal encephalopathy with suspected hypoxic ischaemic encephalopathy (NESHIE), Epigenetics, Hypoxia-inducible factor-1 alpha (HIF-1α), Foetal programming.

## Abstract

Neonatal encephalopathy with suspected hypoxic ischaemic encephalopathy (NESHIE) is a neurological disorder caused by oxygen deprivation and limited blood flow to a neonate’s brain. Although various antenatal and perinatal factors have been identified, their precise role in NESHIE pathogenesis remains unclear. The pathophysiology involves multiple molecular pathways that can be explored using a multi-omics approach, including epigenetics. Epigenetics involves heritable changes in gene expression without altering the DNA sequence, encompassing chemical modifications to DNA and histone proteins, as well as changes mediated by non-coding RNAs (ncRNAs). These epigenetic changes regulate gene expression and can be influenced by environmental factors, offering crucial insights into gene regulation and disease mechanisms. This review examines the role of epigenetic mechanisms in NESHIE, focusing on the modulation of hypoxia-inducible factor-1 alpha (HIF-1α) and ncRNA during hypoxic conditions. Additionally, epigenetic-mediated foetal programming may shed light on how maternal and antenatal risk factors contribute to NESHIE susceptibility. Understanding these epigenetic signatures could advance biomarker discovery and the development of novel therapeutic strategies for NESHIE.

## Background

Neonatal encephalopathy (NE) occurs in late preterm or term new-born infants. It is characterised by a disturbed neurological function that manifests as decreased muscle tone and reflexes, decreased level of consciousness, seizures, and respiratory depression [1–4]. This condition can lead to severe consequences for affected neonates including cerebral palsy, epilepsy, visual, hearing, intellectual impairment, and death [5]. While NE has various causes, hypoxic-ischaemic (HI) injury is the most frequently documented cause of NE. It is termed hypoxic-ischaemic encephalopathy (HIE) [1, 2, 6, 7], or neonatal encephalopathy with suspected hypoxic ischaemic encephalopathy (NESHIE). The latter acronym will be used throughout as it appropriately describes the condition [8].

NESHIE, as the name suggests, results from inadequate cerebral oxygen supply (hypoxia) and restricted cerebral blood flow (ischaemia) during the intrapartum or peripartum period [3, 4]. Classified by severity according to the modified Sarnat grading system into mild, moderate or severe NESHIE [9, 10], the outcomes vary with infants in the mild category generally fully recovering while those with moderate-to-severe NESHIE developing long-term motor and/or cognitive impairments [11–13]. In addition, mortality risk escalates with severity to 60% for new-borns with severe NESHIE [11, 12], making birth asphyxia the fifth leading cause of under-five deaths globally [14]. However, lower–middle-income countries (LMICs) face a higher incidence of NESHIE (23–265 per 1 000 live births) compared to high-income countries (13–17 per 1 000 live births) [5, 6], with most cases being graded as moderate to severe [10, 15–18]. The elevated incidence of NESHIE with increased severity in LMICs poses a significant concern, particularly in regions like South Africa [17, 18].

The pathophysiology of NESHIE involves well-defined molecular pathways and cellular processes that can be analysed by adopting a “multi-omics” approach (genomics, epigenomics, transcriptomics, proteomics, and metabolomics) [19]. Recent studies have delved into several of these aspects with regard to NESHIE, but epigenetics represents an arm that remains underexplored. Epigenetics examines heritable changes in gene expression that occur without alterations to the underlying DNA sequence [20, 21]. It encompasses chemical modifications to DNA or histone proteins and regulation executed by non-coding RNAs [22]. These epigenetic alterations critically control gene expression and can be influenced by environmental factors, hence offering insights into disease mechanisms and gene regulation [23, 24].

Epigenetics has emerged as a pivotal element in the cellular response to hypoxia, with hypoxia-inducible factor 1 alpha (HIF-1α) postulated under epigenetic regulation [25]. Furthermore, non-coding RNAs are expressed in hypoxic conditions, suggesting their involvement in adaptive cellular processes [26, 27]. Studies have, therefore, emphasised the potential role of epigenetic programming in influencing outcomes after hypoxic injury [28–30]. Epigenetic changes may explain how maternal and antenatal risk factors contribute to the susceptibility of NESHIE and its sequelae. Unravelling the epigenetic landscape associated with NESHIE could contribute to our understanding of its molecular pathogenesis and variation in patient outcomes and offer potential biomarkers for diagnosis and prognosis that enhance monitoring and treatment strategies.

### Neonatal encephalopathy with suspected hypoxic ischaemic encephalopathy (NESHIE)

#### Causes and risk factors

The causative pathways leading to NESHIE are complex, and while the exact cause is not always identified, perinatal asphyxia is recognised as the significant contributor [31]. Various peripartum or intrapartum risk factors have been implicated in inducing asphyxia, resulting in HI injury to the neonate’s brain due to inadequate placental perfusion and impaired gaseous exchange. These risk factors include sentinel events or delivery complications (i.e. HI events), such as placental abruption, uterine rupture, umbilical cord prolapse/avulsion, prolonged second stage of labour, shoulder dystocia, abnormal foetal heart rate and tight nuchal cord [4, 13, 32–35]. However, these events are limited to a minority (< 10%) of affected neonates, particularly in LMICs [10, 36], indicating a more diverse aetiology.

Antenatal and maternal risk factors for NESHIE include nulliparity, gestation > 41 weeks, advanced maternal age (> 35), urinary tract infection (UTI) during pregnancy, intrauterine growth restriction, maternal hypertensive disorders and chorioamnionitis [33–35]. Some NESHIE cases may present with both sentinel events and antenatal complications [34], illustrating the multifactorial nature of this condition. Additional risk factors, though less commonly documented, include maternal viral illness, obesity, gestational diabetes, smoking, alcohol consumption, and illicit drug use [6, 37, 38]. However, the exact role of these factors in NESHIE pathogenesis remains unclear.

The severity of NESHIE has been suggested to depend on the degree of the HI event, additional risk factors, and the individual neonatal response. Hypoxic injury is theoretically classified into two major categories: acute and chronic [28], also sometimes referred to as acute profound and chronic intermittent. However, unless there is a sentinel event, the extent and duration of hypoxia cannot be directly quantified in an affected infant [1]. This underscores the need for biomarkers to delineate the nature of the HI injury in neonates with NESHIE.

### Diagnostics

NESHIE is diagnosed based on a combination of clinical and metabolic criteria. This includes low Apgar scores (< 5) at 5–10 min as the neonates may present with bradycardia, poor respiratory effort, hypotonia, decreased alertness, weak or absent cry, abnormal skin colour, and a cord blood pH of < 7 or an early infant blood gas with a base deficit of ≥ 12 mmol/L indicating metabolic acidosis [4, 13, 31, 39]. Diagnosis also considers HI events before or during labour and multi-system organ dysfunction involving the heart, liver and/or kidneys, provided that other causes are excluded [1, 4]. Other tools such as brain imaging tests and neurophysiological monitoring are valuable during the early assessment of infants suspected to have NESHIE. The latter is performed by amplitude integrated electroencephalography (aEEG) that can monitor brain function at the bedside [40, 41]. Magnetic resonance imaging (MRI) is a sensitive and specific imaging modality for assessing NESHIE and can provide a guide to the potential timing of a cerebral insult [4, 31]. Based on brain injury patterns, the extent of HI injury, which correlates with outcomes, can also be inferred, thus assisting in establishing the severity of NESHIE [31, 42, 43]. While this may be a powerful tool in the diagnosis and prognosis prediction of NESHIE, unstable neonates may not be able to endure transportation for a brain MRI nor the duration of the scanning process [43]. Moreover, MRI scans cannot always be conducted on affected infants in poorly resourced settings such as in LMICs given the high number of patients that require such scans [41]. Clinicians, therefore, often rely on other clinical assessment tools such as the Thompson and modified Sarnat scoring systems.

The modified Sarnat scoring system, initially introduced by Sarnat and Sarnat [9] in 1976, defines the clinical spectrum of NESHIE into three stages based on certain distinguishing features as described in Table 1. Affected infants can progress from mild to moderate or severe encephalopathy over the 72-h post-HI injury [10]. This scoring system thus aids in monitoring the progression of neurological dysfunction over time in infants with NESHIE. On the other hand, the Thompson score predicts early neurodevelopmental outcomes of infants with NESHIE by evaluating nine clinical signs related to central nervous system function [44] (Table 2). This numerical scoring system was developed by Thompson and colleagues [44] in 1997 and ranges from 0 to 22 with 0 considered normal nervous system function and higher values implying increasingly poorer function. The Thompson score relates to the modified Sarnat score such that neonates with a Thompson score of 1–6 are considered to have mild NESHIE, 7–14 to have moderate NESHIE and 15–22 to have severe NESHIE [44, 45]. While the parameters of both the modified Sarnat and Thompson grading systems are clear, there are limitations. Both rely heavily on the attending neonatologist’s/paediatrician’s interpretation of the clinical findings that may vary, thus introducing a degree of subjectivity [46]. Biomarker identification is therefore crucial and may result in more accurate and reliable diagnosis and outcome prediction in infants with NESHIE, including those that are critically ill.

**Table 1:** Stages of neonatal encephalopathy as described by Sarnat & Sarnat [8]

Assessment	Stage 1: Mild encephalopathy	Stage 2: Moderate encephalopathy	Stage 3: Severe encephalopathy
Level of consciousness	hyper alert	lethargic	stuporous
Neuromuscular control			
Muscle tone	normal	mild hypotonia	flaccid
Posture	mild distal flexion	strong distal flexion	intermittent decerebration
Stretch reflexes	overactive	overactive	decreased or absent
Segmental myoclonus	present	present	absent
Complex reflexes			
Suck	weak	weak or absent	absent
Moro	strong; low threshold	weak; incomplete; high threshold	absent
Oculovestibular	normal	overactive	weak or absent
Tonic neck	slight	strong	absent
Autonomic function			
Pupils	mydriasis	miosis	variable; often unequal; poor light reflex
Heart rate	tachycardia	bradycardia	variable
Bronchial and salivary secretions	sparse	profuse	variable
Gastrointestinal motility	normal or decreased	increased diarrhoea	variable
Seizures	none	common; focal or multifocal	uncommon (excluding decerebration)
Electroencephalogram findings	normal (awake)	early: low-voltage continuous delta & theta. late: periodic pattern (awake)	early: periodic pattern with iso-potential phases. late: totally iso-potential

**Table 2:** Thompson scoring system as described by Thompson et al*.* [42]

Sign	Score			
	0	1	2	3
Tone	normal	hyper	hypo	flaccid
Level of consciousness	normal	hyper alert; stare	lethargic	comatose
Fits	none	infrequent (< 3/day)	frequent (> 2/day)	
Posture	normal	fisting, cycling	strong, distal flexion	decerebrate
Moro	normal	partial	absent	
Grasp	normal	poor	absent	
Suck	normal	poor	Absent ± bites	
Respiration	normal	hyper	brief apnoea	IPPV
Fontanelle	normal	full, not tense	tense	

IPPV: intermittent positive pressure ventilation

### Treatment strategies

Therapeutic hypothermia (TH) is the standard treatment worldwide for neonates diagnosed with moderate-to-severe NESHIE. Cooling typically begins within the first 6 h of life and core body temperature is reduced to 33.5 ± 0.5 ºC for 72 h. [47, 48] There are two methods of cooling, namely selective head cooling and whole-body cooling. While both methods are equally effective, whole-body cooling is recommended as it is less expensive and easier to operate, involving cool packs and/or cooling blankets [43, 47]. The neuroprotective mechanism of TH aims to reduce cerebral cellular metabolism, which in turn reduces the inflammatory response and minimises brain injury [49]. A Cochrane review in 2013 reported that TH reduces the risk of death or major disability up to 18 months of age by 26% in neonates with moderate-to-severe NESHIE [50]. Interestingly, the multicentre randomised HELIX trial contradicted these findings claiming that TH failed to decrease death or disability up to 18 months of age in LMICs but heightens the risk of death alone [51]. Furthermore, clinical trials have investigated the effects of initiating TH at different time points of life and for a longer duration on neurodevelopmental outcomes [13, 43, 52]. Despite efforts to improve the therapeutic effectiveness of this treatment, only infants with NESHIE who meet certain physiological and neurological criteria (outlined in [48]) are cooled. The exclusion criteria for TH stipulate that neonates with NESHIE who are older than 6 h, have a birth weight < 2 kg, are preterm (gestational age < 36 weeks), have major life-threatening cardiovascular or respiratory system abnormalities, or major congenital malformations may not undergo cooling [47, 48]. For these neonates, only supportive care is available, which entails maintaining adequate ventilation, blood pressure and glucose levels to prevent secondary brain injury [43]. Anticonvulsant medications may also be administered to control seizures commonly observed in neonates with NESHIE [53]. However, enough cooling equipment may not always be available for infants who qualify for TH, which is a challenge faced in many LMICs [18], stressing the need for alternative treatment interventions.

A search is currently underway to identify additional agents that might complement TH and further improve outcomes in infants with NESHIE. Various pharmacological agents have demonstrated neuroprotective properties in animal models and/or clinical trials and have been suggested as potential adjuvant drugs [11, 54, 55] (Table 3). Babbo and colleagues [56] recently reviewed the biological treatment modalities investigated in clinical trials, with a particular focus on their implications for LMICs. Given the uncertainty around TH’s benefits and the challenge of treatment resources in LMICs, the authors emphasise the need for more accessible and effective stand-alone treatment options in these settings. Ideally, ongoing research should strive towards developing or identifying an agent that is inexpensive, easy to store and administer, has a broad therapeutic effect and is accessible to treat infants with NESHIE worldwide.

**Table 3:** Classes of neuroprotective drugs investigated in combination with therapeutic hypothermia to treat infants with NESHIE. [53] Adapted from Table 2 in Ovcjak et al*.*

Drug class	Drug name	Mechanism of action
γ-aminobutyric acid (GABA) receptor antagonists	Phenobarbital, Topiramate	Minimises neuronal excitability
N-methyl-d-aspartate (NMDA) receptor antagonists	Magnesium sulphate, Xenon gas	Decreases extracellular calcium influx and accumulation of toxic metabolites
Neurogenic and angiogenic agents	Erythropoietin, Darbepoetin alpha	Induces neurogenesis and angiogenesis which stimulates remodelling and repair of cell functioning
Stem cells	Umbilical cord blood cells (includes mesenchymal stem cells)	Promotes neural and vascular repair; upregulates growth factors
Glucocorticoids	Hydrocortisone	Suppresses inflammatory and immune responses
Antioxidants	Melatonin, Allopurinol	Reduces oxidative stress within the cell by neutralising free radicals

### Pathophysiology

**Fig. 1:**
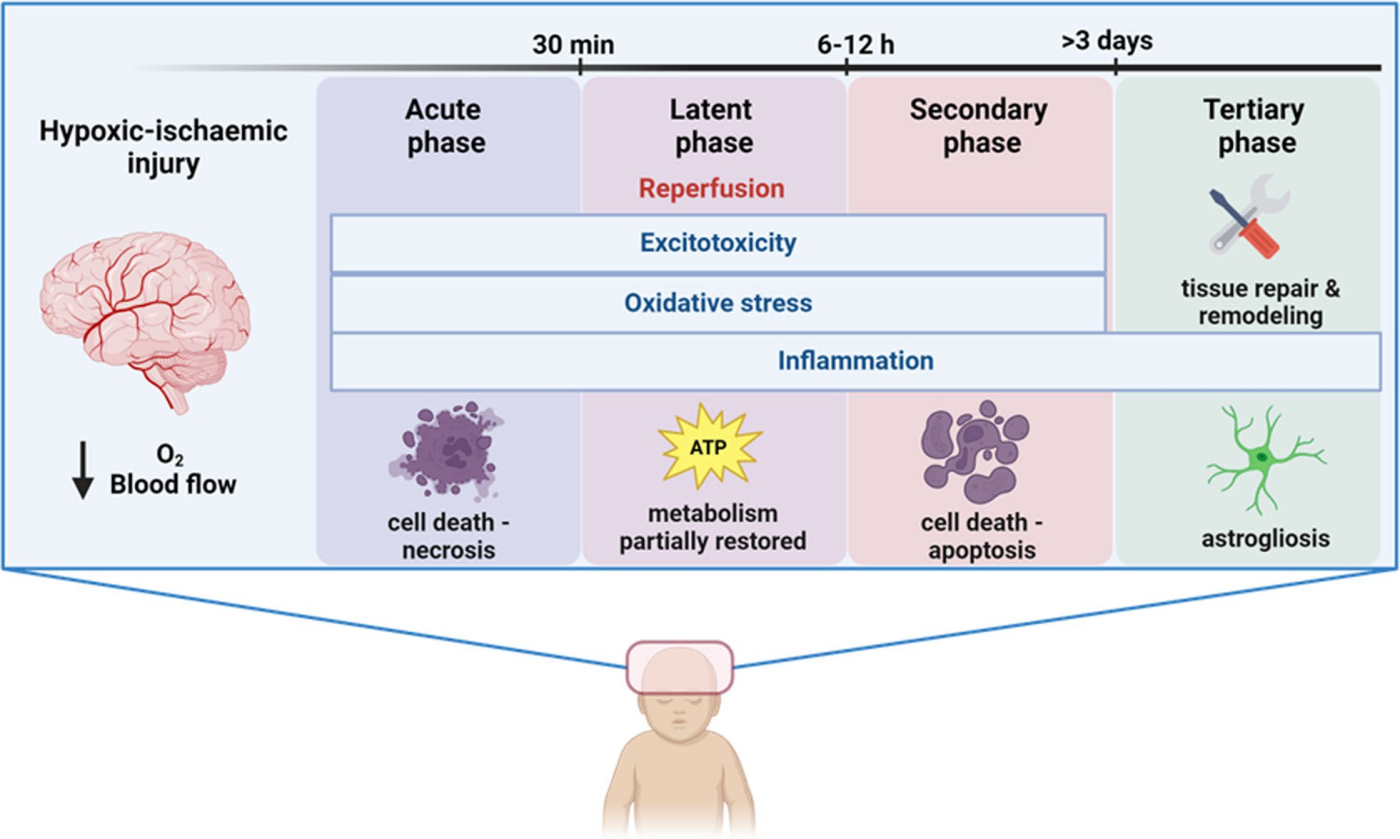
Overview of the pathophysiological features of NESHIE, adapted from Douglas-Escobar *et al* [43] and Pedroza-García et al. [11]. Image was created by P Mistry using Biorender (https://www.biorender.com/)

NESHIE pathophysiology involves a complex cascade of events that occur in response to the HI insult that culminate in the clinical presentation of brain injury. These events evolve and can be divided into four phases (Fig. 1). These phases are underpinned by three central pathogenic mechanisms, namely excitotoxicity, oxidative stress and inflammation.

The foetal brain is highly dependent on aerobic respiration to produce energy in the form of adenosine triphosphate (ATP) [57]. During an HI insult, the foetal brain is deprived of oxygen and glucose, which leads to impaired ATP production [13, 32]. This acute primary energy failure causes brain cells (e.g. astrocytes, microglial cells, and neurons) to switch to anaerobic respiration, accumulating lactic acid [11, 32]. The energy deficit also results in the dysfunction of crucial transmembrane ion pumps, leading to the influx and build-up of intracellular water, sodium (Na^+^) and calcium (Ca^2^^+^) [11, 32]. This triggers membrane depolarisation in neuronal cells, which stimulates the release of glutamate, an excitatory amino acid, from presynaptic neurons [11, 58]. The release of glutamate initiates excitotoxicity, which involves the overactivation of postsynaptic N-methyl-d-aspartate (NMDA), α-amino-3-hydroxy-5-methyl-4-isoxazolepropionic acid (AMPA) and kainate receptors allowing the influx of additional Ca^2^^+^ into neural cells [11, 58]. Consequently, cellular swelling and mitochondrial dysfunction with resultant cell necrosis or apoptosis can occur [32]. In addition, reactive oxygen species (ROS) cannot be effectively eliminated by antioxidant enzymes due to the interrupted cellular metabolism [57]. Nitric oxide (NO) synthase is also activated upon influx of excess Ca^2+^, which generates NO free radicals [57, 58]. Together, the surplus of ROS and free radicals causes oxidative damage to which neonatal neurons are particularly vulnerable [32] .

Parallel to the cellular responses of the brain, the innate immune system is triggered within minutes after an HI injury. This response is typically characterised by the activation of microglia and astrocytes that release pro-inflammatory cytokines, chemokines and matrix metalloproteinases (MMPs), leading to the breakdown of the blood–brain barrier (BBB) [57, 59, 60]. As a result, peripheral leukocytes infiltrate the brain, which is usually protected from systemic immune responses [57]. The influx of these inflammatory cells exacerbates neuroinflammation, causing further neuronal injury and secondary brain injury [59, 60]. Activated microglia, as well as peripheral leukocytes, also release ROS and NO, adding to the oxidative stress [60] .

Following acute primary energy failure, reperfusion, along with medical intervention (if available), ushers in the latent phase approximately 30–60 min after the HI event [43, 61]. Cerebral metabolism is partially restored during this phase, and many treatment interventions target this therapeutic window [13, 58]. It has been suggested that the duration of the latent period varies inversely to the severity of the HI insult, i.e. the harsher the insult, the shorter the latent period [62]. Failure to effectively restore homeostasis during this period promotes the onset of secondary energy failure, such as in the case of moderate-to-severe injury [43]. Like primary, secondary energy failure results from excitotoxicity, oxidative stress and inflammation [32, 58]. However, these persistent processes lead to delayed neuronal death predominantly by apoptosis as opposed to cell necrosis [60]. Seizures are also characteristic of this period [43]. Given the prolonged and irreversible neuronal injury and death, secondary energy failure is described as the major cause of the neurological sequelae observed in NESHIE cases [63] .

A tertiary chronic injury phase can occur after secondary energy failure that continues for months and involves chronic inflammation, tissue repair and remodelling, and astrogliosis that further contributes to neuronal cell death and brain injury [43, 60] .

Based on the phases of injury described, it is evident that a multitude of molecular pathways and cellular processes drive the pathophysiology of NESHIE. It is critical to gain a deeper understanding of these molecular aspects not only to enhance our knowledge on the pathogenesis of NESHIE, but also for the development of effective treatment and preventative measures. Potential population-specific predisposing factors/conditions that may contribute to the high prevalence of NESHIE in LMICs need to be considered. In efforts to address these concerns, the novel biology-based “multi-omics” approach (genomics, epigenomics, transcriptomics, proteomics, and metabolomics) has recently gained attention in the field of NESHIE-related research [19]. The advantage of this approach is that it allows researchers to examine various layers of biological information simultaneously, thereby increasing the depth and breadth of NESHIE research and providing a holistic understanding of the mechanisms involved. Additionally, multi-omics analyses allow for the identification of critical molecular pathways and signatures, from which therapeutic targets, as well as biomarkers can be leveraged for NESHIE diagnosis, prognosis and measurement of treatment response [19]. Several authors have reviewed and summarised the genes/genetic variants [19, 64], RNA molecules [19], proteins [19, 65], and metabolites [19, 65–67] identified as potential candidate biomarkers. These candidate biomarkers were identified after applying relevant “omics” technology to various biological samples isolated from infants with NESHIE. Montaldo and colleagues [68] also investigated, using transcriptomics, whether differences in gene expression profiles of infants with NESHIE might explain the discrepancy in hypothermic neuroprotection observed in the HELIX trial. However, studies elucidating the epigenetic profile of infants with NESHIE are lacking.

## Epigenetics

### Definition

Conrad Waddington first introduced the concept of “epigenetics” in the early 1940s as ‘the branch of biology which studies the causal interactions between genes and their products which bring the phenotype into being’ [69]. Over the years, this definition has matured into the definition accepted in literature today as ‘the study of heritable changes in gene expression that occur without modulating the underlying DNA sequence’ [20, 21]. As demonstrated by the ‘epigenetic landscape’ published by Conrad Waddington, epigenetic changes regulate gene activity by determining when and where genes are activated or suppressed [70, 71]. This regulation ensures cell-type-specific gene expression, thus playing a crucial role in cellular differentiation and dictating the identity and distinct biological function of cells in the body [71, 72]. Although most of the epigenome is persistent for this purpose, it is also considered to be highly dynamic, influenced by biological processes and environmental factors that potentially either induce or reverse epigenetic marks [23, 24] .

### Major mechanisms

**Fig. 2:**
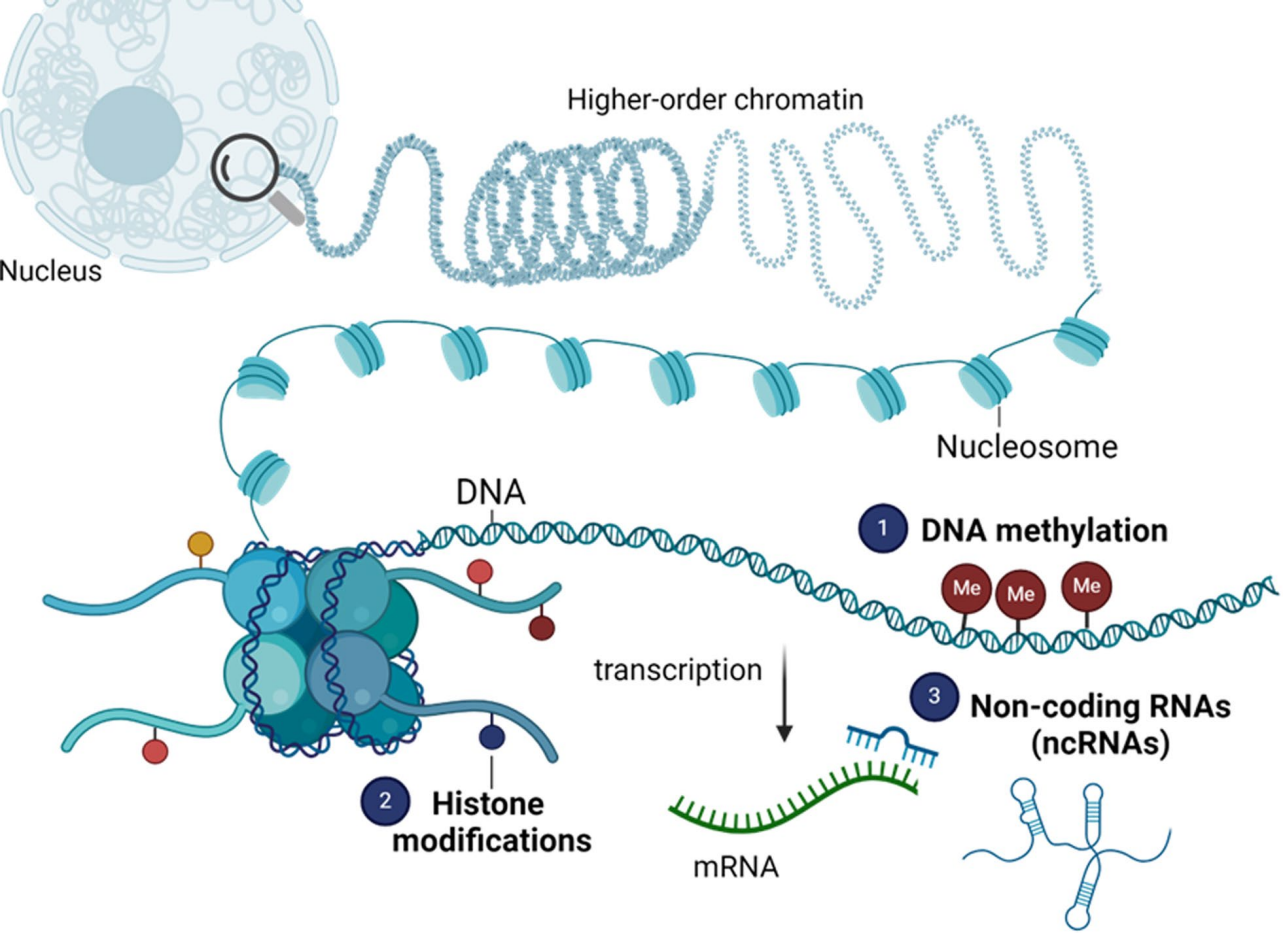
Molecular mechanisms involved in epigenetic gene control, adapted from Wu et al. [71]. Image was created by P Mistry using Biorender (https://www.biorender.com/)

Epigenetic modifications include chemical changes to DNA or histones, as well as changes mediated by non-coding RNAs. The following section describes the mechanisms that confer these alterations and their effects on gene expression at various levels (Fig. 2). It should be noted that while each epigenetic mediator is an independent cellular event and often discussed as such, there is a degree of crosstalk between them.

#### DNA methylation

DNA methylation refers to the addition of a methyl group to the 5’ carbon position of cytosine bases within the DNA molecule. This covalent modification is catalysed by DNA methyltransferases (DNMTs) that transfer the methyl group from S-adenylmethionine to cytosine converting it to 5-methylcytosine [73, 74]. In human cells, five DNMTs have been identified: DNMT1, DNMT2, DNMT3A, DNMT3B and DNMT3L. They are classified into two groups: de novo DNMTs and maintenance DNMTs. The latter is facilitated by DNMT1 alone, while DNMT3A and DNMT3B mediate de novo methylation [73–75]. In contrast, DNMT2 and DNMT3L do not possess catalytic activity but have sequence conservation with the other DNMTs [74]. DNA demethylation can also occur either passively through the inhibition of DNMT1 maintenance methylase or actively through the action of ten-eleven translocation (Tet) enzymes Tet1, Tet2 and Tet3 [74] .

Methylation occurs predominantly in the cytosine of cytosine-phosphate-guanine (CpG) dinucleotides which may cluster along the genome, where they are referred to as CpG islands [22]. CpG islands are enriched in promoter regions of 60–70% of genes, making these elements prone to DNA methylation [74, 76]. Hypermethylation of promoter regions induces transcriptional silencing through several suggested mechanisms. DNA methylation can directly impede the binding of transcriptional factors, thereby prohibiting the initiation of transcription [77]. Alternatively, DNA methylation is recognised by methyl-CpG-binding proteins (MBPs), such as methyl-CpG-binding protein 2 (MeCP2) [74, 77]. The exact mechanism by which MBPs repress transcription is not fully understood, but it has been suggested that they recruit repressor complexes [77]. DNA methylation patterns can be studied using molecular biology techniques that analyse methylation levels at specific loci or on a genome-wide scale (reviewed in [78]). Bisulfite sequencing is the gold-standard technology for studying DNA methylation at single-base-pair resolution and can be applied at a gene or global genome level [79] .

#### Histone modifications

Histone modifications refer to the chemical changes on the N-terminus of histone proteins that occur following the process of translation, i.e. post-translational modifications. Some of the most frequently documented modifications include acetylation, methylation, phosphorylation, ubiquitination and sumoylation. Like DNA methylation, histone methylation is catalysed by histone methyltransferases (HMTs), whereas acetylation occurs through histone acetyltransferases (HATs) and can be reversed under the action of histone deacetylases (HDACs) [24, 80] .

Histone modifications are responsible for regulating transcription by modulating chromatin structure. In brief, DNA is tightly packaged in the nucleus of eukaryotic cells around histone proteins, forming a complex called a nucleosome [80, 81]. Each nucleosome consists of two copies of each histone subunit, namely histone 2A (H2A), H2B, H3, and H4, that are arranged in an octameric core with approximately 147 bp of DNA wrapped around it [81]. The linker protein, histone 1 (H1), connects the nucleosomes along with linker DNA that varies in length [81]. Histones are rich in positively charged amino acids, such as lysine (K) and arginine (R), that interact with the negatively charged phosphate groups of DNA, ensuring a tight chromatin configuration. However, post-translational modifications to specific K or R histone residues can alter the charge of the histones, resulting in either a weakened or strengthened interaction between histones and DNA [24]. A weakened interaction relaxes DNA, allowing access and binding of transcription machinery to DNA elements, which subsequently results in transcriptional activation [28, 29]. Conversely, a strengthened interaction condenses DNA preventing the accessibility of transcription machinery. Different histone modifications have diverse effects on the transcriptional status depending on the amino acid and the position of the residue it occurs at (Table 4) [82]. Altogether, the location of the histone in relation to the gene and the combination of histone modifications determine chromatin accessibility [29, 83]. Although chromatin immunoprecipitation and immunoblotting methods are employed to study histone modifications at specific sites [24], the assay for transposase-accessible chromatin with sequencing (ATAC-seq) is suggested to profile the global chromatin accessibility of a genome amongst others [83, 84] .

**Table 4:** Histone modifications and their effect on transcription. [73] Adapted from Zhao & Garcia

Modification	Histone sites	Transcriptional effect
Acetylation of lysine	H3 positions 2,4,9,14,18 & 56 H4 positions 5,8,12,16,20. H2A & H2B	Activation
Methylation of lysine	H3 positions 4,36,79 H3 positions 9,27; H4 positions 12,20	Activation Repression
Methylation of arginine	H3 positions 2,17,26 H4 position 3	Activation
Phosphorylation of serine/threonine	H3 positions 3,10,11,28 H4 position 1 H2A & H2B	Activation
Ubiquitination of lysine	H2B position 120 H2A position 119	Activation Repression
Sumoylation of lysine	H2B position 5 H2A position 126	Repression

#### Non-coding RNAs (ncRNAs)

Only 2% of the human genome codes for proteins, while the remainder of the genome is transcribed into non-coding RNAs (ncRNAs) [85]. Non-coding RNAs are divided into infrastructural ncRNAs that are constitutively expressed (ribosomal, transfer, and small nuclear RNAs, etc.) and regulatory ncRNAs that participate in epigenetic regulation of gene expression [86]. Based on their length, regulatory ncRNAs are classified into small ncRNAs (18–200 nucleotides) and long ncRNAs (lncRNAs; > 200 nucleotides) [73, 87]. Recently, new classes of ncRNAs have been introduced, termed circular RNAs (circRNAs) [88], promoter-associated RNAs (PARs), and enhancer RNAs (eRNAs) [86, 89]. Small ncRNAs are further categorised into micro-RNAs (miRNAs), small interfering RNAs (siRNAs) and piwi-interacting RNAs (piRNAs). The biosynthesis of each of the different ncRNAs classes is distinct. Still, it involves the action of Drosha and Dicer enzymes to cleave the double-stranded RNA precursor molecules, such as those found in siRNAs and miRNAs [90]. Wei *et al* [87] provide a comprehensive overview of the processing of each of the most prominent ncRNAs.

Unlike DNA methylation and histone modifications, ncRNAs can modulate expression at multiple levels. Non-coding RNAs can control the initiation of transcription either at the level of the gene (by guiding DNMT enzymes to specific genomic regions) or chromatin (by recruiting histone-modifying enzymes such as HDACs to histone sites) [91–93]. ncRNAs also mediate post-transcriptional gene regulation by interacting with messenger RNA (mRNA), either preventing translation or promoting degradation [91–93]. However, the mechanisms by which each of the major ncRNAs executes gene control at the different levels vary (Table 5).

**Table 5:** Summary of the characteristics and mechanism of gene control carried out by the major ncRNAs

ncRNA class		Length (nt)	Mechanism of gene control
Small non-coding RNAs	Micro-RNA (miRNA)	20–24	Bind primarily to the 3’ untranslated region (UTR) of target mRNAs facilitating post-transcriptional gene suppression in two ways: • Perfect complementarity resulting in deadenylation of the mRNA and subsequent degradation by exonucleases • Partial complementarity wherein ribosome recruitment is blocked thus repressing translation Interact with key DNA methylation and histone-modifying enzymes thus regulating transcription
	Small interfering RNA (siRNA)	20–24	siRNA strand is loaded into the RNA-induced silencing complex (RISC) which then binds to the complementary target mRNA strand, within the UTR, and mediates post-transcriptional gene silencing through: • Endonucleolytic cleavage of the strand leading to its degradation • Association of RISC-siRNA complex with mRNA hinders the binding of translation machinery thereby inhibiting translation
	Piwi-interacting RNA (piRNA)	24–31	Mainly regulate transposable elements in the germline by: • Associating with Piwi family proteins, a subfamily of Argonaute proteins, to produce piRNA-induced silencing complexes (piRISCs). These piRISCs recognise and bind complementary target transposable element transcripts, inducing endonucleolytic cleavage of the transcript and subsequent suppression of transposon activity • Mediating transcriptional silencing of transposable elements by guiding Piwi proteins to their genomic loci and promoting a condensed chromatin configuration, preventing the binding of transcriptional machinery
Long non-coding RNA (lncRNA)		> 200	Modulate gene expression at various levels due to their ability to localise to the nucleus or cytoplasm where they exert distinct functions: • Influence the recruitment of transcriptional activators or repressors to specific loci, thereby controlling the transcription of nearby genes • Serve as decoys or guides for miRNAs sequestering them away from their target to other mRNA strands, hence implementing post-transcriptional regulation • Recruit histone-modifying enzymes that alter the histone code and the resultant chromatin configuration/accessibility

### Inheritance

#### Mitosis

Epigenetic patterns are mitotically stable because they can be transmitted from somatic cells to their daughter cells [20, 94]. This faithful inheritance ensures that gene expression states that govern cell identity and function are maintained during cell division [95]. During DNA replication, parental strands are separated, and new unmethylated daughter strands are synthesised resulting in an asymmetrical methylation pattern referred to as hemi-methylation [24, 96]. Maintenance DNMTs (DNMT1) recognise these hemi-methylated DNA sites and methylate the unmodified cytosine, thereby restoring the DNA methylation pattern [97]. Similarly, when nucleosomes are reassembled after the replication fork, the old, modified histones are equally distributed between the DNA strands along with new naïve histones [98]. Histone-modifying enzymes and histone chaperones such as the Polycomb repressive complex 2 have a dual read-writer function that enables them to identify (‘read’) histone modifications on old histones and establish (‘write’) the same modification on the naïve histones where relevant [96, 99]. However, ncRNAs are not directly inherited via mitosis per se, given that RNA is synthesised de novo in each cell cycle. Instead, certain ncRNAs play a role in establishing DNA methylation and histone modifications, which are faithfully transmitted and hence indirectly influence gene expression across cell divisions [96] .

#### Meiosis

Meiosis is a specialised form of cell division by which gametes (sperm and oocytes in humans) are generated [100]. Unlike mitosis, meiosis occurs solely in the gonads of sexually reproducing organisms, i.e. the testes of males and the ovaries of females in humans [100]. To understand the epigenetic dynamics during meiosis, it is crucial to appreciate the epigenetic transitions in gamete development. Gametogenesis is the formation of mature gametes from precursor germ cells, which encompasses meiosis [101]. Precursor germ cells stem from primordial germ cells (PGCs) that begin to develop in the early embryo during gastrulation from the epiblast and migrate to the gonadal ridges (precursors to the gonads) [102, 103]. Following migration, PGCs are said to undergo genome-wide loss of DNA methylation, mediated by Tet enzymes, that triggers extensive chromatin remodelling [103–105]. There is a particular loss of repressive histone methylation (H3K9me3 and H3K27me3) and active chromatin marks (H3K9ac), but numerous marks do persist [103, 105]. Once sex determination has been initiated, DNA methylation patterns in PGCs are gradually restored via de novo methylation along with histone post-translational modifications [104, 106]. Although uncertainty surrounds the extent and timing of this event, the re-establishment of epigenetic signatures is crucial for the onset of meiosis [104]. These epigenetic signatures are then propagated during meiosis I and meiosis II through the mechanisms of replicative transmission described for mitosis [96]. However, murine models have suggested that the levels of H3K9me3 and H3K27me3 may be altered during chromosome pairing and recombination at prophase of meiosis I, but the purpose of this remains unknown [104]. Around puberty, germ cells complete both divisions of meiosis, whereby they become mature gametes [101]. Interestingly, during the final stage of maturity, histone proteins are replaced by nuclear transition proteins in sperm cells that are later replaced by protamine proteins [101, 105]. These proteins are associated with an overall condensed chromatin architecture that is reported to protect DNA from mutagens and free radicals as mature sperm no longer divide [107]. As opposed to sperm, mature oocytes maintain their nucleosomal histones throughout [104]. Non-coding RNAs also play a role in germ cell development, especially piRNAs that guards genomic stability and participate in DNA methylation reprogramming of PGCs [105, 108]. Apart from the dynamic epigenetic changes that take place under homeostatic conditions during gametogenesis, environmental cues can also introduce epigenetic changes in germ cells [96, 105]. These epigenetic changes accumulate in the germline and are maintained throughout gametogenesis via the mechanisms described [96, 104, 109]. But the impact of these changes on gametogenesis and whether they disturb the process remains poorly understood.

#### Transgenerational inheritance

Transgenerational inheritance of epigenetic information requires the manifestation of epigenetic changes in the germ line that are permanent [20, 95, 110]. Having said this, shortly after fertilisation, the zygote undergoes a wave of epigenetic reprogramming involving the erasure of DNA methylation from both the maternal and paternal genomes [111, 112]. The purpose of this erasure is to form totipotent embryonic cells that have the capacity to differentiate into all the specialised cell types for embryonic development [111, 112]. To implement the epigenetic marks required for embryonic development and cellular differentiation, the inner cell mass and trophoblast start to acquire de novo DNA methylation under the action of DNMT3A and DNMT3B [112]. The DNA methylation landscape continues to change during embryonic development all the way through adulthood such that studies associate the extent of DNA methylation with ageing [28, 113] .

The fact that the epigenome of an early embryo is reset to a baseline level begs the question as to whether epigenetic marks present in germ cells can be passed down to the next generation. The Dutch Famine of 1944–1945 is a well-documented scenario that provided the first direct evidence of epigenetic transgenerational effects [114–116]. Offspring of males and females who were exposed to the famine around the time of conception were observed to have an increased incidence of metabolic disease amongst others [116]. A similar consequence was observed for the progeny of pregnant mothers during the famine as well as the generation after their progeny (F2 generation) [116]. The individuals from families who were exposed to the famine were documented to have a lower methylation status of insulin-like growth factor II (IGF-2) that affected their metabolism [114, 115]. Thus, exposure to the famine likely altered the epigenome of the germ cells of men and women before conception that were inherited by their offspring [114, 117]. Likewise, through in utero exposure to the famine, the epigenome of unborn offspring was altered, as were the offspring’s germ cells due to the presence of developing PGCs [114, 117] .

Two potential models for the transgenerational inheritance of DNA methylation have been proposed: the escapee model and the reconstruct model. As the name suggests, the escapee model refers to regions that can resist DNA methylation reprogramming events during PGC development and post-fertilisation [109]. This is typically the case with imprinted genes that are differentially marked according to the parent of origin, meaning that their expression is determined by whether they are inherited from the mother or father [109, 118]. These genes are flanked by or include repetitive DNA sequences called imprinting control regions (ICRs) that sustain the DNA methylation state of imprinted genes during epigenetic reprogramming events [119]. Reconstructive transmission proposes that DNA methylation patterns may be erased but can be inherited through reconstruction by a secondary epigenetic signal [96, 109]. Transcription factors or ncRNAs may act as secondary signals but only upon minor environmental stimulation that matches that of the previous generation [96, 109] .

### Foetal programming

Although epigenetic transgenerational inheritance is an important contributor to an individual’s epigenome, environmental exposure also plays a crucial role. In the case of new-borns, the *in utero* environment is considered the first environment they are exposed to. Because this period represents a critical window of embryonic and foetal development, environmental exposures can have long-lasting effects on the health and physiology of the offspring [20, 24]. This phenomenon is known as foetal programming. It is influenced almost purely by the lifestyle and habits of the mother during gestation, including diet, stress, and exposure to toxins, among others [20, 24, 120]. The concept of foetal programming originated from the observations made by Barker et al. that highlighted the long-term impact of the in utero environment on the health outcomes of the offspring [121]. This later expanded to the Developmental Origins of Health and Disease hypothesis, a framework that encompasses the idea of foetal programming and extends beyond the antenatal period to include early infancy and childhood [122, 123]. In line with this hypothesis, substantial evidence indicates that because the foetus is directly exposed to the same lifestyle factors as the mother in utero*,* these factors may induce epigenetic changes (to both somatic and germ cells) that potentially increase the risk of certain health outcomes later in the offspring’s life such as obesity, diabetes and neurodevelopmental disorders [124, 125]. It is, therefore, of utmost importance to consider maternal antenatal factors, especially in the instance of disease.

### Epigenetics in the context of NESHIE

#### Hypoxia-inducible factor 1 alpha (HIF-1α)

**Fig. 3:**
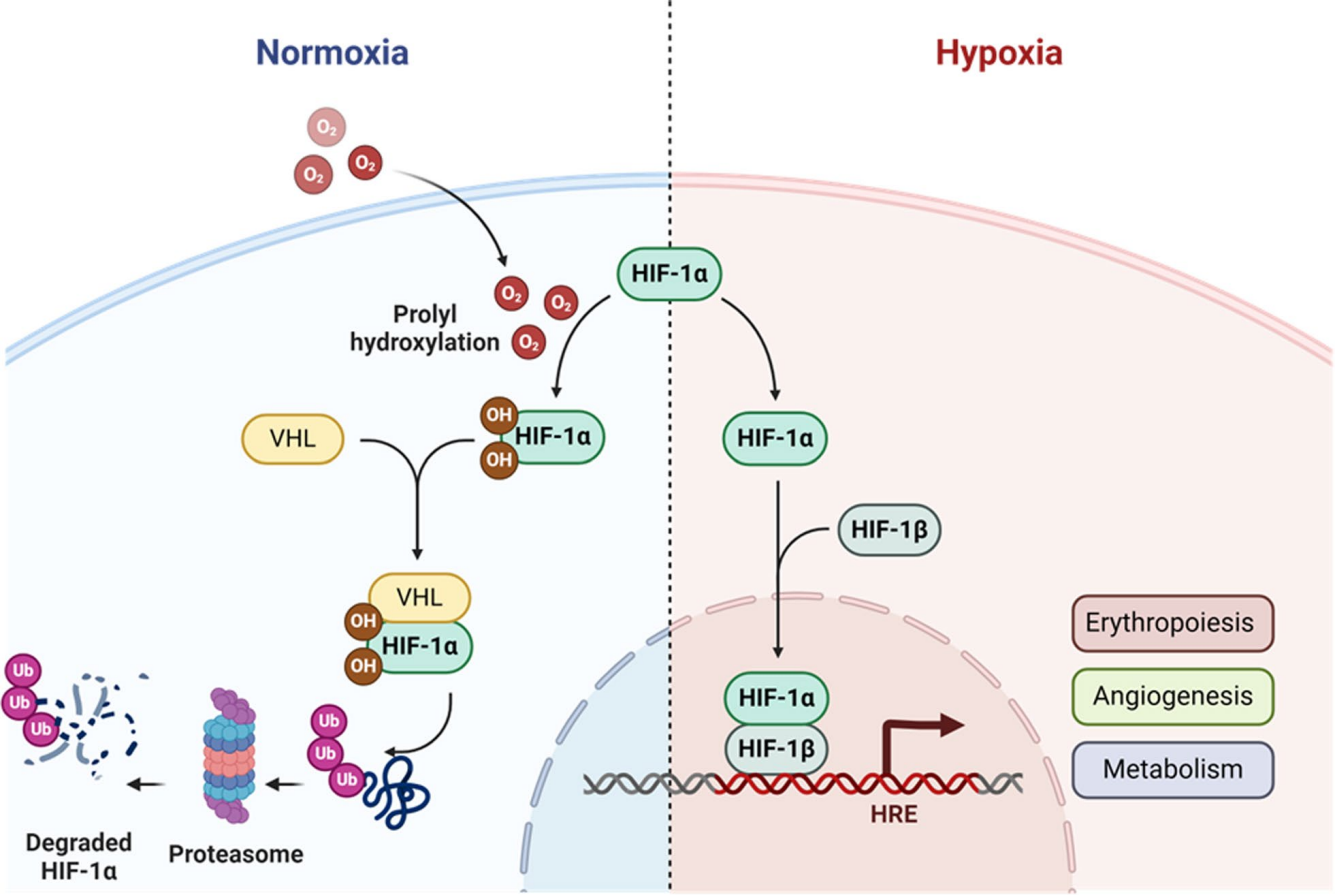
The hypoxia-inducible factor pathway under normoxic and hypoxic conditions, adapted from Suresh et al. [126]. Image was created by P Mistry using Biorender (https://www.biorender.com/). (HIF: hypoxia-inducible factor; VHL: von Hippel–Lindau; HRE: hypoxia response element; ub: ubiquitin)

The body’s canonical response to hypoxia involves hypoxia-inducible factor 1 (HIF-1), a transcription factor with two subunits: HIF-1α and HIF-1β [126]. While HIF-1β is stable and constitutively expressed independent of oxygen levels, HIF-1α is the master mediator of the cellular response to hypoxia, being tightly regulated by oxygen status [127–129]. (Fig. 3) Under normoxic conditions, HIF-1α is hydroxylated at proline residues by oxygen-sensitive prolyl hydroxylases (PHDs) [130, 131]. Hydroxylated HIF-1α is then recognised and marked for ubiquitination by an E3 ubiquitin ligase, the von Hippel–Lindau protein (pVHL) complex, for ubiquitin-mediated proteasomal degradation, hence the short half-life of HIF-1α [131–133]. However, under hypoxic stress, PHDs are inhibited due to insufficient oxygen as a cofactor [127]. This results in the stabilisation, accumulation and translocation of HIF-1α to the nucleus where it heterodimerises with HIF-1β [131, 132, 134, 135]. The HIF-1 complex then binds to hypoxia response elements (HREs) of genes that participate in the adaptive cellular response to hypoxia, including processes such as erythropoiesis, angiogenesis and metabolism [135, 136] .

Compared to ambient conditions, the *in utero* environment is naturally hypoxic and gradually becomes less hypoxic as pregnancy progresses, particularly during the last weeks of gestation [137]. Further impairing oxygen levels, such as during an HI event, subjects the foetus to hypoxic stress during which the HIF-1α signalling pathway is expected to be triggered. Indeed, murine models have demonstrated the activation of the HIF-1α response under conditions of cerebral ischaemia, similar to NESHIE [134, 138, 139]. However, the involvement of HIF-1α in either promoting protective mechanisms or causing injury is a subject of debate, given the detrimental effects of chronic HIF-1α expression [133, 140, 141] .

Key role players of the HIF-1α signalling pathway have been suggested to be under epigenetic regulation. The HIF-1α promoter is rich in CpG islands, making it prone to DNA methylation that would suppress the expression of the resultant protein and its cellular abundance [26, 142, 143]. Similarly, HREs contain CpG sites that, when methylated, reduce the binding affinity of the heterodimerised HIF-1 to these sites, as observed with increased HRE methylation levels in the 3’ erythropoietin (EPO) enhancer [144] .

Histone modifications provide another layer of epigenetic regulation, wherein HIF-1α engages with crucial epigenetic modifiers. HIF-1α interacts with HDAC1 as well as with components of polycomb repressive complexes like suppressor of zeste 12 (SUZ12) [145, 146]. Through these interactions, HIF-1α is stabilised, and chromatin is remodelled into transcriptionally repressive or permissive states in a context-dependent manner. This remodelling regulates the accessibility of HIF-1α to DNA, thereby modulating the activation of genes required for the hypoxic response. Under hypoxic conditions, HIF-1α also transcriptionally induces members of the Jumonji family of histone demethylases by binding to their HRE, which removes repressive histone methyl marks and further facilitates the expression of hypoxia-responsive genes [143, 147]. In parallel, the DNA dioxygenase Tet1 is transcriptionally induced in a HIF-1-dependent manner and facilitates the accumulation of 5-hydroxymethylcytosine—the first step in active DNA demethylation—at HREs [148]. This modification contributes to chromatin relaxation, thereby enabling HIF-1α binding and the expression of hypoxia-responsive genes.

Collectively, these diverse epigenetic modifications—DNA methylation, histone post-translational modifications, chromatin remodelling and DNA hydroxymethylation—coordinate to regulate HIF-1α expression and function. They enable precise temporal and spatial control of gene expression patterns critical for cellular adaptation to hypoxic stress. Several authors have reviewed the potential role of epigenetics in dictating the outcomes after hypoxic injury in the instance of NESHIE, focusing primarily on brain development and neurological outcomes [28–30]. However, much of this mechanistic insight stems from in vitro cell line studies or animal models as described here. The specific epigenetic landscape in response to hypoxia in infants with NESHIE remains largely unexplored due to the lack of human studies, representing an important frontier for future research.

#### ncRNAs—miRNA

In addition to DNA methylation, miRNAs have been implicated in the HIF-1α response under hypoxic conditions. Experiments conducted on murine-derived tumour models and cell lines identified HIF-1α mRNA transcripts as the target of miR-17–92 clusters, miR-20b and miR-199a miRNA molecules that thereby regulate the protein abundance of HIF-1α [149–151]. On the other hand, miRNAs such as miR-23, miR-24, miR-26, miR-107 and miR-210 are downstream targets of the HIF-1 complex that are induced during hypoxic conditions [26, 30, 152]. Interestingly, miR-210 is well described in literature as the ‘master of hypoxia-induced miRNA’ because it has been consistently shown to be upregulated during hypoxia in various cell types [26, 27]. The HIF-1 complex binds to the highly conserved HRE in the promoter region of miR-210 but nuclear factor κB (NFκb) can also drive the expression of miR-210 in response to hypoxia [27, 153]. During hypoxia, miR-210 functions to regulate mitochondrial metabolism, stimulate angiogenesis as well as progression of the cell cycle, thereby affecting cell fate [154]. Whitehead and colleagues [155] reported the elevation of miR-210, amongst others, in the maternal blood of pregnancies complicated by foetal hypoxia. Other studies investigated the miRNA profile of umbilical cord blood (UCB) from infants with NESHIE using microarray technology or targeted qPCR and revealed the downregulation of miR-374a, miR-181b, miR-199a and miR-376c [156–158]. Levels of miR-210 and miR-374a along with S100B protein and neuron-specific enolase (NSE) enzyme demonstrated highly accurate diagnostic and prognostic value with levels corresponding to HIE severity and neurological assessment scores [159]. Alternatively, the extent of miR-374a downregulation correlated with the degree of hypoxic insult, such that combining miR-374a and miR-210 is suggested to hold prognostic value [29]. While these miRNAs present as promising non-invasive biomarkers, a deeper understanding of the ncRNA signature of hypoxia in infants with NESHIE could assist in identifying additional biomarkers to complement those currently under investigation. Furthermore, human studies are needed that adopt an unbiased, global approach to analysing the ncRNA landscape in larger cohorts (n > 150) compared to previous studies.

### Pathogenic mechanisms

While HIF-1α plays a central role in the cellular response to hypoxia and its epigenetic regulation is well described in the literature, it is important to recognise the epigenetic regulation of other molecules involved in NESHIE pathogenesis. Inflammatory mediators such as NF-κB and pro-inflammatory cytokines are regulated epigenetically through DNA methylation and histone modifications [160, 161]. For example, histone methyltransferase EZH2 and the demethylase JMJD3 control the transcription of inflammatory genes and neurotrophic factors, ultimately shaping neuroinflammation and repair following hypoxic injury [160]. Notably, in murine models of intermittent neonatal hypoxia the antioxidant enzyme superoxide dismutase 2 (SOD2) was shown to be epigenetically silenced via DNA hypermethylation at a single CpG site near its transcription start site [162]. This modification resulted in sustained oxidative stress, suggesting a potential mechanism that exacerbates oxidative damage following HI injury. Similarly, epigenetic regulation of growth factors such as EPO influences cellular responses to hypoxia: while HIF-1 and HIF-2 drive EPO expression, DNA methylation and histone modifications can restrict gene accessibility, thereby silencing its expression in certain contexts [163–165]. Moreover, murine models of neonatal HIE demonstrate that DNA and histone methylation modulate genes controlling apoptotic signalling and inflammatory responses [145], with EZH2 further regulating stress-response pathways under hypoxia [166]. This suggests that dysregulated epigenetic control may drive maladaptive apoptosis and inflammation, contributing to the severity and poor outcomes observed in NESHIE. Together, these epigenetically regulated molecules highlight the multifaceted nature of the hypoxic response in NESHIE and underscore the urgent need for human studies to characterise these epigenetic alterations.

### RNA methylation

N^6^-methyladenosine (m^6^A) is one of the most well-studied and abundant post-transcriptional modifications in eukaryotic mRNA [167]. Functionally, RNA methylation regulates several aspects of RNA metabolism including stability, splicing, localisation, translation efficiency and degradation [167]. Similar to DNA methylation, this methylation is catalysed by “writer” methyltransferase complexes such as methyltransferase-like 3 (METTL3) and reversed by “eraser” demethylases [168, 169]. In the context of hypoxic stress, m^6^A RNA methylation regulates cellular responses by modulating the stability and translation of transcripts involved in stress adaptation and inflammation [167], processes highly relevant to neonatal HI brain injury. Changes in RNA methylation patterns can also influence neural development and cell fate decisions [170], suggesting a potentially critical role in NESHIE pathophysiology. Exploring the emerging field of epitranscriptomics may provide an important addition to multi-omics approaches in NESHIE research, with the potential to reveal novel molecular mechanisms and therapeutic targets that extend current insights.

### Antenatal risk factors and foetal programming

As mentioned, NESHIE presents with a multifactorial aetiology, with some cases potentially involving maternal and antenatal environmental factors. While the exact role of these factors in NESHIE pathogenesis remains unclear, epigenetic mechanisms may be the link between the two. Considering foetal programming, there is great interest in uncovering how maternal habits during sensitive periods of development might influence epigenetics and subsequently the developing foetus. This is particularly relevant in the context of neurodevelopmental disorders [171, 172].

#### Maternal habits (alcoholism, smoking and illicit drug use)

Maternal habits such as alcohol use and smoking during pregnancy have been reported to be associated with NESHIE [173, 174]. Dumont and colleagues reported that foetal alcohol exposure in a murine NESHIE model resulted in significant motor impairment, suggesting that maternal alcoholism in a NESHIE setting may worsen outcomes. Various animal models have shown the epigenetic changes in the placenta, UCB [175, 176] and brain tissue [177] induced by alcohol exposure and their role in foetal alcohol spectrum disorders (FASDs). However, studies investigating epigenetic alterations in these tissues from human pregnancies are unfortunately rare. This may be due to the challenge of recruiting a large enough cohort of mothers who consume alcohol during pregnancy or the level of honesty when reporting the use of alcohol during pregnancy [171]. Nevertheless, investigating the epigenetic signature induced by alcohol exposure and how it relates to NESHIE is pertinent particularly in settings such as South Africa, where there is a high prevalence of both NESHIE and FASDs, with FASD occurring at a rate of 29 to 290 per 1 000 live births [178] .

Epigenome-wide association studies have revealed that in utero prenatal smoke exposure results in a global DNA hypomethylation in the placenta [179, 180] and UCB [181] of new-borns. These methylation changes resulted in the dysregulated expression of genes involved in brain development, oxidative stress, and hypoxia signalling, particularly the induction of HIF-1α [179, 180]. The authors attributed utero-placental insufficiency commonly observed in tobacco-exposed foetuses to the aberrant functioning of the hypoxia-signalling pathway [180]. Given that utero-placental insufficiency is a potential cause of chronic foetal hypoxia, these findings implicate maternal smoking in the onset of NESHIE. Lastly, studies analysing the impact of maternal illicit drug use are scarce, with only one documented case report. Maddox and colleagues [182] linked the development of NESHIE in a new-born male to the heroin use of the mother. They proposed that heroin often leads to low blood pressure, significantly compromising cerebral blood flow and contributing to the development of NESHIE.

#### Maternal infections and inflammation

During pregnancy, the maternal immune system transitions to a state of partial immune suppression to prevent immune rejection of foetal antigens [183, 184]. While this may be an immunological adaptation, women are more susceptible to infections during this time [184]. Urinary tract infections (UTIs) are among the most prevalent bacterial infections in pregnant women. They are associated with adverse maternal and foetal outcomes including intrauterine growth restriction, low birth weight, preterm delivery and chorioamnionitis [185, 186]. Interestingly, UTIs have also been identified as an antenatal risk factor for mental retardation, childhood epilepsy as well as NESHIE [34, 185]. Other than UTIs, pregnant mothers commonly present with respiratory infections, yeast infections and sexually transmitted infections (STIs), many of which are associated with pregnancy and foetal complications [183, 184, 187, 188]. A South African study reported a high prevalence of STIs during pregnancy, the majority of which were asymptomatic and occurred in human immunodeficiency virus (HIV)-positive women [189]. Chronic immune activation and inflammation are key features of HIV infection, which, when coupled with the immune response to STIs, expose the foetus to exacerbated inflammatory conditions [190]. A murine model has provided evidence of inflammation during prenatal life, inducing epigenetic changes in offspring, suggesting that epigenetics may be involved in the manifestation of adverse outcomes associated with infections during pregnancy [191]. Parker and colleagues [34] hypothesised that the maternal inflammation induced in response to infections such as UTI during pregnancy may potentially trigger a foetal inflammatory cascade that reduces foetal tolerance to HI stressors, thus worsening the neurological sequelae of NESHIE. The role of maternal infections and inflammation in NESHIE is not yet established, but the increase in circulating cytokines in infants with NESHIE suggests this potential association [192]. Therefore, it may be of interest to explore if exposure to maternal infections and inflammation triggers epigenetic changes in maternal and foetal tissues, potentially affecting gene expression patterns and cellular responses in infants with NESHIE.

#### Maternal co-morbidities

Maternal co-morbidities such as gestational diabetes, obesity and hypertensive disorders have emerged as potential risk factors for NESHIE [37, 38, 193]. A retrospective study conducted in Indonesia found that neonates born to mothers with preeclampsia, a hypertensive disorder defined by increased blood pressure and proteinuria during pregnancy, had a higher incidence of NESHIE alongside other neonatal complications [194]. It has been suggested that increased maternal blood pressure can cause a reduction in the flow of oxygen to the foetus, leading to NESHIE, but epigenetic changes may also play a role. Epigenetic mechanisms have been implicated in the pathophysiology of preeclampsia, with multiple studies examining the DNA methylation signatures in the cord blood and placental tissue of neonates born to preeclamptic mothers [195–198]. Studies that adopted epigenome-wide analyses reported contradicting overall DNA methylation levels in the UCB of these infants, but hypomethylation was the predominant finding [196]. Of note, candidate-gene approaches reported a lower methylation level of two genes, solute carrier family 2 member 1 (SLC2A1) and basic helix-loop-helix family member e40 (BHLHE40), in the placental tissue of pregnancies complicated by severe preeclampsia [196]. Given that both genes are under HIF-1-mediated expression [199, 200], these findings suggest that maternal preeclampsia may induce DNA methylation changes that influence the hypoxic response of placental tissue. In addition, miR-20a and miR-20b were observed to be upregulated in preeclampsia in placental tissue and function in conjunction to downregulate HIF-1α [197]. The increase in these miRNA molecules in a preeclamptic pregnancy may thus interfere with the adaptive response to hypoxia, dampening processes such as angiogenesis in the placenta [197]. Should gestational hypoxia occur, the placenta may not be able to adapt and nourish the foetus, leading to insufficient oxygenation and the potential onset of NESHIE in the foetus.

Maternal gestational diabetes increases the risk of metabolic abnormalities. It is associated with an elevated risk of death or unfavourable short-term neonatal outcomes in infants with NESHIE, such as longer duration of ventilation, prolonged hospital stay and the need for tube feeding [37]. Aberrant DNA methylation patterns observed during pregnancy complications such as maternal diabetes have been suggested as a potential mediator of in utero foetal programming that results in the adverse outcomes observed in the offspring [201]. Several studies have investigated [198, 202, 203] and reviewed [201, 204] the DNA methylation patterns in UCB and placental tissue from pregnancies with gestational diabetes. Dias *et al.* [201] and Haertle *et al.* [202] highlighted the increased methylation of the hypoxia-inducible factor 3 alpha (HIF-3α) gene in UCB of infants born to mothers with gestational diabetes compared to non-diabetic controls. Like other members of the HIF family (HIF-1α and HIF-2α), HIF-3α is upregulated under hypoxic conditions but has a dual function [205, 206]. First, it acts as a negative regulator of HIF-1α and HIF-2α by competing with these subunits for the binding of the HIF-1β subunit, resulting in the repression of HIF-1- and HIF-2-mediated gene expression [205]. Second, it may function as a transcription factor activating the expression of specific target genes involved in the hypoxic adaptive response, some of which overlap with HIF-1α [207, 208]. The increased methylation of the HIF-3α gene in infants born to pregnancies with diabetic mothers suggests the suppression of this gene. Considering the biological functions of HIF-3α, these neonates may be faced with uncontrolled HIF-signalling and/or an inadequate hypoxic response should an HI event occur, which may underlie the unfavourable outcomes associated with NESHIE.

Gestational overweight and obesity are well described to be associated with an increased risk of pregnancy complications such as preterm birth, congenital defects, stillbirth, and preeclampsia as well as an increased risk for the development of cardiometabolic disease in adulthood of the offspring [209, 210]. Retrospective studies have also indicated that neonates of overweight/obese mothers [Body mass index (BMI) ≥ 30] were significantly more likely to be diagnosed with NESHIE with increased severity, suggesting that maternal obesity confers an additional risk of NESHIE [38, 211]. Although the pathophysiologic mechanisms behind these findings have not been elicited, a growing body of evidence suggests that obesity, through epigenetic mechanisms, alters the offspring’s phenotype, impacting the risk of disease [38, 209, 212]. Compared to normal-weight pregnancies, placental tissue from overweight/obese pregnancies presents with a higher level of global methylation [198], whereas UCB has significantly less methylated cytosines [212, 213]. In addition, pathway enrichment analysis of differentially methylated genes in UCB revealed the aryl hydrocarbon receptor nuclear translocator (ARNT) transcription factor as one of the genes with a marked decline in methylation status [213]. The ARNT transcription factor is also known as HIF-1β [213], implying that maternal obesity may have ramifications in the neonatal response to hypoxia; however, this remains to be investigated in the context of NESHIE.

### Potential epigenetic therapeutic interventions

Targeting epigenetic mechanisms has great potential in the management of NESHIE, as has been demonstrated in other disease contexts. Small-molecule inhibitors of DNMTs and HDACs have already advanced into clinical trials for various neurological and oncological conditions, highlighting their ability to modify epigenetic states [214, 215]. These agents can reverse aberrant DNA methylation and histone modification patterns, thereby restoring gene expression profiles essential for brain repair and neuroprotection. In NESHIE specifically, HDAC inhibitors such as sodium butyrate, valproic acid and trichostatin A represent the only epigenetic interventions currently reported to reduce neuronal injury in preclinical rodent models of neonatal HI brain injury [216]. Beyond neuroprotection, treatment with these inhibitors also attenuated inflammation, oxidative stress and apoptosis while stimulating neurogenesis and exerting anti-inflammatory effects [216, 217]. To facilitate the identification of additional therapeutic targets in neonates with NESHIE, a comprehensive mapping of the epigenetic landscape—including DNA methylation patterns and ncRNA profiles—is needed. In addition to small molecules, emerging genome- and epigenome-editing technologies, such as CRISPR-based epigenetic editors, also offer the potential for precise, tissue-specific, and tunable modulation of epigenetic marks, which could be tailored for NESHIE interventions. Despite these advances, translation to the clinic remains limited, and the efficacy, safety and delivery strategies of epigenetic therapies in this vulnerable population remain to be established.

## Conclusions

NESHIE is a multifaceted neurological disorder in new-borns that can lead to long-term neurodevelopmental disabilities or even death if not promptly diagnosed and managed. As reviewed here, by elucidating the molecular responses to hypoxia and their modulation by maternal health, we could gain valuable insight into the pathophysiology of NESHIE. In addition, epigenetic alterations can potentially serve as biomarkers for risk assessment and prognosis prediction in affected neonates. Moving forward, further research is warranted to uncover the complex interactions between genetic, environmental and epigenetic factors in the development and progression of NESHIE. Investigating the epigenetic profile of blood from infants with NESHIE may provide clues to the interaction between these factors. Moreover, by analysing samples obtained directly from patients, we could better represent the epigenetic landscape in response to hypoxia and other related pathways than the speculations made from animal models and cell lines. Findings from ex vivo studies have translational potential in the clinical setting that can pave the way for more effective diagnosis and management strategies for neonates with NESHIE.

## Data Availability

No datasets were generated or analysed during the current study.

## Abbreviations

aEEG: Amplitude-integrated electroencephalography
AMPA: α-Amino-3-hydroxy-5-methyl-4-isoxazolepropionic acid
ARNT: Aryl hydrocarbon receptor nuclear translocator
ATAC-seq: Assay for transposase-accessible chromatin with sequencing
ATP: Adenosine triphosphate
BBB: Blood–brain barrier
BHLHE40: Basic helix-loop-helix family member e40
BMI: Body mass index
BSL: Biosafety level
circRNAs: Circular RNAs
CpG: Cytosine-phosphate guanine
DNMTs: DNA methyltransferases
EPO: Erythropoietin
eRNAs: Enhancer RNAs
FASDs: Foetal alcohol spectrum disorders
GABA: γ-Aminobutyric acid
H2A: Histone 2A
HATs: Histone acetyltransferases
HDACs: Histone deacetylases
HI: Hypoxic-ischaemic
HIE: Hypoxic-ischaemic encephalopathy
HIF-1: Hypoxia-inducible factor 1
HIF-1α: Hypoxia-inducible factor 1 alpha
HIF-3α: Hypoxia-inducible factor 3 alpha
HIV: Human immunodeficiency virus
HMTs: Histone methyltransferases
HREs: Hypoxia response elements
ICRs: Imprinting control regions
IGF-2: Insulin-like growth factor II
K: Lysine
LMICs: Low- and middle-income countries
lncRNAs: Long non-coding RNAs
MBPs: Methyl-CpG-binding proteins
MeCP2: Methyl-CpG-binding protein 2
METTL3: Methyltransferase-like 3
miRNAs: Micro-RNAs
MMPs: Matrix metalloproteinases
MRI: Magnetic resonance imaging
mRNA: Messenger RNA
ncRNAs: Non-coding RNAs
NE: Neonatal encephalopathy
NESHIE: Neonatal encephalopathy with suspected hypoxic ischaemic encephalopathy
NFκb: Nuclear factor κB
NMDA: N-methyl-d-aspartate
NO: Nitric oxide
NSE: Neuron-specific enolase
PARs: Promoter-associated RNAs
PGCs: Primordial germ cells
PHDs: Prolyl hydroxylases
piRISCs: PiRNA-induced silencing complexes
piRNAs: Piwi-interacting RNAs
pVHL: Von Hippel–Lindau protein
R: Arginine
RISC: RNA-induced silencing complex
ROS: Reactive oxygen species
siRNAs: Small interfering RNAs
SLC2A1: Solute carrier family 2-member 1
SOD2: Superoxide dismutase 2
STIs: Sexually transmitted infections
SUZ12: Suppressor of zeste 12
Tet: Ten-eleven translocation
TH: Therapeutic hypothermia
TNCs: Total nucleated cells
UCB: Umbilical cord blood
UTI: Urinary tract infection
UTR: Untranslated region

## References

[1] Aslam S, Strickland T, Molloy EJ. Neonatal encephalopathy: need for recognition of multiple etiologies for optimal management. Front Pediatr. 2019;7:142. 10.3389/fped.2019.00142. 31058120 10.3389/fped.2019.00142 PMC6477286

[2] Sandoval Karamian AG, Mercimek-Andrews S, Mohammad K, et al. Neonatal encephalopathy: etiologies other than hypoxic-ischemic encephalopathy. Semin Fetal Neonatal Med. 2021;26(5):101272. 10.1016/j.siny.2021.101272. 34417137 10.1016/j.siny.2021.101272

[3] Molloy EJ, Branagan A, Hurley T, et al. Neonatal encephalopathy and hypoxic–ischemic encephalopathy: moving from controversy to consensus definitions and subclassification. Pediatr Res. 2023;94(6):1860–3. 10.1038/s41390-023-02775-z. 37573378 10.1038/s41390-023-02775-z PMC13366674

[4] ACOG Task Force on Neonatal Encephalopathy. 2014 Executive summary: neonatal encephalopathy and neurologic outcome, second edition. Obstet Gynecol. 123 (4): 896–901 10.1097/01.AOG.0000445580.65983.d2 10.1097/01.AOG.0000445580.65983.d2 24785633

[5] Ristovska S, Stomnaroska O, Danilovski D. Hypoxic ischemic encephalopathy (HIE) in term and preterm infants. Prilozi. 2022;43(1):77–84. 10.2478/prilozi-2022-0013. 10.2478/prilozi-2022-0013 35451288

[6] Kurinczuk JJ, White-Koning M, Badawi N. Epidemiology of neonatal encephalopathy and hypoxic–ischaemic encephalopathy. Early Hum Dev. 2010;86(6):329–38. 10.1016/j.earlhumdev.2010.05.010. 20554402 10.1016/j.earlhumdev.2010.05.010

[7] Sell E, Munoz FM, Soe A, et al. Neonatal encephalopathy: case definition & guidelines for data collection, analysis, and presentation of maternal immunisation safety data. Vaccine. 2017;35(48 Pt A):6501–5. 10.1016/j.vaccine.2017.01.045. 29150055 10.1016/j.vaccine.2017.01.045 PMC5710979

[8] Horn AR, Pillay S, Velaphi SC, et al. Neonatal encephalopathy due to suspected hypoxic–ischaemic encephalopathy. World J Pediatr. 2025. 10.1007/s12519-025-00952-0. 10.1007/s12519-025-00952-0 40742666

[9] Sarnat HB, Sarnat MS. Neonatal encephalopathy following fetal distress: a clinical and electroencephalographic study. Arch Neurol. 1976;33(10):696–705. 10.1001/archneur.1976.00500100030012. 987769 10.1001/archneur.1976.00500100030012

[10] Padayachee N, Ballot DE. Outcomes of neonates with perinatal asphyxia at a tertiary academic hospital in Johannesburg, South Africa. S Afr J Child Health. 2013;7:89–94.

[11] Pedroza-García KA, Calderón-Vallejo D, Quintanar JL. Neonatal hypoxic-ischemic encephalopathy: perspectives of neuroprotective and neuroregenerative treatments. Neuropediatrics. 2022;53(6):402–17. 10.1055/s-0042-1755235. 36030792 10.1055/s-0042-1755235

[12] Finer NN, Robertson CM, Richards RT, et al. Hypoxic-ischemic encephalopathy in term neonates: perinatal factors and outcome. J Pediatr. 1981;98(1):112–7. 10.1016/s0022-3476(81)80555-0. 7452386 10.1016/s0022-3476(81)80555-0

[13] Shankaran S. Neonatal encephalopathy: treatment with hypothermia. J Neurotrauma. 2009;26(3):437–43. 10.1089/neu.2008.0678. 19281415 10.1089/neu.2008.0678 PMC2828322

[14] Bryce J, Boschi-Pinto C, Shibuya K, et al. WHO estimates of the causes of death in children. Lancet. 2005;365(9465):1147–52. 10.1016/s0140-6736(05)71877-8. 15794969 10.1016/S0140-6736(05)71877-8

[15] Namusoke H, Nannyonga MM, Ssebunya R, et al. Incidence and short term outcomes of neonates with hypoxic ischemic encephalopathy in a peri urban teaching hospital, Uganda: a prospective cohort study. Matern Health Neonatol Perinatol. 2018;4:6. 10.1186/s40748-018-0074-4. 29556412 10.1186/s40748-018-0074-4 PMC5840790

[16] Simiyu IN, McHaile DN, Katsongeri K, et al. Prevalence, severity and early outcomes of hypoxic ischemic encephalopathy among newborns at a tertiary hospital, in northern Tanzania. BMC Pediatr. 2017;17(1):131. 10.1186/s12887-017-0876-y. 28545428 10.1186/s12887-017-0876-y PMC5445334

[17] Horn AR, Swingler GH, Myer L, et al. Defining hypoxic ischemic encephalopathy in newborn infants: benchmarking in a South African population. J Perinat Med. 2013;41(2):211–7. 10.1515/jpm-2012-0107. 23096100 10.1515/jpm-2012-0107

[18] Nakwa FL, Sepeng L, van Kwawegen A, et al. Characteristics and outcomes of neonates with intrapartum asphyxia managed with therapeutic hypothermia in a public tertiary hospital in South Africa. BMC Pediatr. 2023;23(1):51. 10.1186/s12887-023-03852-2. 36721127 10.1186/s12887-023-03852-2 PMC9890846

[19] Rasineni GK, Panigrahy N, Rath SN, et al. Diagnostic and therapeutic roles of the “omics” in hypoxic-ischemic encephalopathy in neonates. Bioengineering. 2022. 10.3390/bioengineering9100498. 10.3390/bioengineering9100498 PMC9598631 36290466

[20] Bohacek J, Mansuy IM. Epigenetic inheritance of disease and disease risk. Neuropsychopharmacol. 2013;38(1):220–36. 10.1038/npp.2012.110. 10.1038/npp.2012.110 PMC3521963 22781843

[21] Wu C, Morris JR. Genes, genetics, and epigenetics: a correspondence. Science. 2001;293(5532):1103–5. 10.1126/science.293.5532.1103. 11498582 10.1126/science.293.5532.1103

[22] Dupont C, Armant DR. Epigenetics: definition, mechanisms and clinical perspective. Semin Reprod Med. 2009;27(5):351–7. 10.1055/s-0029-1237423. (**Brenner CA**). 19711245 10.1055/s-0029-1237423 PMC2791696

[23] Tompkins JD, Hall C, Chen VC, et al. Epigenetic stability, adaptability, and reversibility in human embryonic stem cells. Proc Natl Acad Sci U S A. 2012;109(31):12544–9. 10.1073/pnas.1209620109. 22802633 10.1073/pnas.1209620109 PMC3411986

[24] Toraño EG, García MG, Fernández-Morera JL, et al. The impact of external factors on the epigenome: in utero and over lifetime. BioMed Res Int. 2016;2016:2568635. 10.1155/2016/2568635. 27294112 10.1155/2016/2568635 PMC4887632

[25] Watson JA, Watson CJ, McCann A, et al. Epigenetics, the epicenter of the hypoxic response. Epigenetics. 2010;5(4):293–6. 10.4161/epi.5.4.11684. 20418669 10.4161/epi.5.4.11684

[26] Ma Q, Xiong F, Zhang L. Gestational hypoxia and epigenetic programming of brain development disorders. Drug Discov Today. 2014;19(12):1883–96. 10.1016/j.drudis.2014.09.010. 25256780 10.1016/j.drudis.2014.09.010 PMC4262616

[27] Chan YC, Banerjee J, Choi SY, et al. miR-210: the master hypoxamir. Microcirculation. 2012;19(3):215–23. 10.1111/j.1549-8719.2011.00154.x. 22171547 10.1111/j.1549-8719.2011.00154.x PMC3399423

[28] Cristancho AG, Marsh ED. Epigenetics modifiers: potential hub for understanding and treating neurodevelopmental disorders from hypoxic injury. J Neurodev Disord. 2020;12(1):37. 10.1186/s11689-020-09344-z. 33327934 10.1186/s11689-020-09344-z PMC7745506

[29] Bustelo M, Barkhuizen M, van den Hove DLA, et al. Clinical implications of epigenetic dysregulation in perinatal hypoxic-ischemic brain damage. Front Neurol. 2020;11:483. 10.3389/fneur.2020.00483. 32582011 10.3389/fneur.2020.00483 PMC7296108

[30] Ma Q, Zhang L. Epigenetic programming of hypoxic–ischemic encephalopathy in response to fetal hypoxia. Prog Neurobiol. 2015;124:28–48. 10.1016/j.pneurobio.2014.11.001. 25450949 10.1016/j.pneurobio.2014.11.001 PMC4272655

[31] Bano S, Chaudhary V, Garga UC. Neonatal hypoxic-ischemic encephalopathy: a radiological review. J Pediatr Neurosci. 2017;12(1):1–6. 10.4103/1817-1745.205646. 28553370 10.4103/1817-1745.205646 PMC5437770

[32] Allen KA, Brandon DH. Hypoxic ischemic encephalopathy: pathophysiology and experimental treatments. Newborn Infant Nurs Rev. 2011;11(3):125–33. 10.1053/j.nainr.2011.07.004. 21927583 10.1053/j.nainr.2011.07.004 PMC3171747

[33] Peebles PJ, Duello TM, Eickhoff JC, et al. Antenatal and intrapartum risk factors for neonatal hypoxic ischemic encephalopathy. J Perinatol. 2020;40(1):63–9. 10.1038/s41372-019-0531-6. 31611618 10.1038/s41372-019-0531-6

[34] Parker S-J, Kuzniewicz M, Niki H, et al. Antenatal and intrapartum risk factors for hypoxic-ischemic encephalopathy in a US birth cohort. J Pediatr. 2018;203:163–9. 10.1016/j.jpeds.2018.08.028. 30270166 10.1016/j.jpeds.2018.08.028

[35] Torbenson VE, Tolcher MC, Nesbitt KM, et al. Intrapartum factors associated with neonatal hypoxic ischemic encephalopathy: a case-controlled study. BMC Pregnancy Childbirth. 2017;17(1):415. 10.1186/s12884-017-1610-3. 29228911 10.1186/s12884-017-1610-3 PMC5725836

[36] Malan R, Jvd L, Kritzinger A, et al. South African neonates with mild and moderate hypoxic-ischaemic encephalopathy. S Afr J Child Health. 2022;16(3):134–8. 10.7196/SAJCH.2022.v16i3.1901.

[37] Laval N, Paquette M, Talsmat H, et al. Exposure to maternal diabetes during pregnancy is associated with aggravated short-term neonatal and neurological outcomes following perinatal hypoxic-ischemic encephalopathy. Am J Perinatol. 2023. 10.1055/a-2053-7904. 10.1055/a-2053-7904 36918161

[38] Monaco-Brown M, Munshi U, Horgan MJ, et al. Association of maternal obesity and neonatal hypoxic-ischemic encephalopathy. Front Pediatr. 2022;10:850654. 10.3389/fped.2022.850654. 35573967 10.3389/fped.2022.850654 PMC9099066

[39] MacLennan AH, Thompson SC, Gecz J. Cerebral palsy: causes, pathways, and the role of genetic variants. Am J Obstet Gynecol. 2015;213(6):779–88. 10.1016/j.ajog.2015.05.034. 26003063 10.1016/j.ajog.2015.05.034

[40] Pu Y, Zhu Z, Yang Q, et al. Significance of amplitude integrated electroencephalography in early stage of neonatal hypoxic-ischemic encephalopathy and cerebral function monitoring in Neonatal Intensive Care Units. Am J Transl Res. 2021;13(8):9437–43. 34540063 PMC8430076

[41] Maphake J, Naidoo H, Coetzee M, et al. The accuracy of the Thompson score in predicting early outcome in neonates with hypoxic ischaemic encephalopathy treated with therapeutic cooling in a tertiary hospital. S Afr Med J. 2023;113(7):35–40. 10.7196/SAMJ.2023.v113i6.220. 10.7196/SAMJ.2023.v113i6.220 37882044

[42] Cabaj A, Bekiesińska-Figatowska M, Mądzik J. MRI patterns of hypoxic-ischemic brain injury in preterm and full term infants—classical and less common MR findings. Pol J Radiol. 2012;77(3):71–6. 10.12659/pjr.883379. 23049586 10.12659/pjr.883379 PMC3447438

[43] Douglas-Escobar M, Weiss MD. Hypoxic-ischemic encephalopathy: a review for the clinician. JAMA Pediatr. 2015;169(4):397–403. 10.1001/jamapediatrics.2014.3269. 25685948 10.1001/jamapediatrics.2014.3269

[44] Thompson CM, Puterman AS, Linley LL, et al. The value of a scoring system for hypoxic ischaemic encephalopathy in predicting neurodevelopmental outcome. Acta Paediatr. 1997;86(7):757–61. 10.1111/j.1651-2227.1997.tb08581.x. 9240886 10.1111/j.1651-2227.1997.tb08581.x

[45] Bhagwani DK, Sharma M, Dolker S, et al. To study the correlation of Thompson scoring in predicting early neonatal outcome in post asphyxiated term neonates. J Clin Diagn Res. 2016;10(11):Sc16–9. 10.7860/jcdr/2016/22896.8882. 10.7860/JCDR/2016/22896.8882 PMC5198415 28050462

[46] Douglas-Escobar M, Weiss MD. Biomarkers of hypoxic-ischemic encephalopathy in newborns. Front Neurol. 2012;3:144. 10.3389/fneur.2012.00144. 23130015 10.3389/fneur.2012.00144 PMC3486976

[47] Lemyre B, Chau V. Hypothermia for newborns with hypoxic-ischemic encephalopathy. Paediatr Child Health. 2018;23(4):285–91. 10.1093/pch/pxy028. 30657134 10.1093/pch/pxy028 PMC6007306

[48] Mosalli R. Whole body cooling for infants with hypoxic-ischemic encephalopathy. J Clin Neonatol. 2012;1(2):101–6. 10.4103/2249-4847.96777. 24027701 10.4103/2249-4847.96777 PMC3743149

[49] Silveira RC, Procianoy RS. Hypothermia therapy for newborns with hypoxic ischemic encephalopathy. J Pediatr (Rio J). 2015;91(6, Supplement 1):S78–83. 10.1016/j.jped.2015.07.004. 26354871 10.1016/j.jped.2015.07.004

[50] Jacobs SE, Berg M, Hunt R, et al. (2013) Cooling for newborns with hypoxic ischaemic encephalopathy. Cochrane Database Syst Rev. 2013;1:cd003311. 10.1002/14651858.CD003311.pub3. 10.1002/14651858.CD003311.pub3 PMC7003568 23440789

[51] Thayyil S, Pant S, Montaldo P, et al. Hypothermia for moderate or severe neonatal encephalopathy in low-income and middle-income countries (HELIX): a randomised controlled trial in India, Sri Lanka, and Bangladesh. Lancet Glob Health. 2021;9(9):e1273–85. 10.1016/S2214-109X(21)00264-3. 34358491 10.1016/S2214-109X(21)00264-3 PMC8371331

[52] Thoresen M, Tooley J, Liu X, et al. Time is brain: starting therapeutic hypothermia within three hours after birth improves motor outcome in asphyxiated newborns. Neonatology. 2013;104(3):228–33. 10.1159/000353948. 24030160 10.1159/000353948

[53] Yozawitz E, Stacey A, Pressler RM. Pharmacotherapy for seizures in neonates with hypoxic ischemic encephalopathy. Paediatr Drugs. 2017;19(6):553–67. 10.1007/s40272-017-0250-4. 28770451 10.1007/s40272-017-0250-4

[54] Parker L, Kenner C. Neuroprotective strategies for hypoxic ischemic encephalopathy. NAINR. 2012;12(1):8–11. 10.1053/j.nainr.2011.12.005.

[55] Ovcjak A, Pontello R, Miller SP, et al. Hypothermia combined with neuroprotective adjuvants shortens the duration of hospitalization in infants with hypoxic ischemic encephalopathy: meta-analysis. Front Pharmacol. 2023. 10.3389/fphar.2022.1037131. 10.3389/fphar.2022.1037131 PMC9853207 36686686

[56] Babbo C, Mellet J, van Rensburg J, et al. Neonatal encephalopathy due to suspected hypoxic ischemic encephalopathy: pathophysiology, current and emerging treatments. World J Pediatr (In press). 2024;20(11):1105. 10.1007/s12519-024-00836-9 PMC11582131 39237728

[57] Greco P, Nencini G, Piva I, et al. Pathophysiology of hypoxic–ischemic encephalopathy: a review of the past and a view on the future. Acta Neurol Belg. 2020;120(2):277–88. 10.1007/s13760-020-01308-3. 32112349 10.1007/s13760-020-01308-3

[58] Albrecht M, Zitta K, Groenendaal F, et al. Neuroprotective strategies following perinatal hypoxia-ischemia: taking aim at NOS. Free Radic Biol Med. 2019;142:123–31. 10.1016/j.freeradbiomed.2019.02.025. 30818057 10.1016/j.freeradbiomed.2019.02.025

[59] Liu F, McCullough LD. Inflammatory responses in hypoxic ischemic encephalopathy. Acta Pharmacol Sin. 2013;34(9):1121–30. 10.1038/aps.2013.89. 23892271 10.1038/aps.2013.89 PMC3764334

[60] Li B, Concepcion K, Meng X, et al. Brain-immune interactions in perinatal hypoxic-ischemic brain injury. Prog Neurobiol. 2017;159:50–68. 10.1016/j.pneurobio.2017.10.006. 29111451 10.1016/j.pneurobio.2017.10.006 PMC5831511

[61] Kleuskens DG, Gonçalves Costa F, Annink KV, et al. Pathophysiology of cerebral hyperperfusion in term neonates with hypoxic-ischemic encephalopathy: a systematic review for future research. Front Pediatr. 2021. 10.3389/fped.2021.631258. 10.3389/fped.2021.631258 PMC7884860 33604320

[62] Iwata O, Iwata S, Thornton JS, et al. “Therapeutic time window” duration decreases with increasing severity of cerebral hypoxia-ischaemia under normothermia and delayed hypothermia in newborn piglets. Brain Res. 2007;1154:173–80. 10.1016/j.brainres.2007.03.083. 17475224 10.1016/j.brainres.2007.03.083

[63] Espinoza MI, Parer JT. Mechanisms of asphyxial brain damage, and possible pharmacologic interventions, in the fetus. Am J Obstet Gynecol. 1991;164(6 Pt 1):1582–9. 10.1016/0002-9378(91)91440-8. 1904682 10.1016/0002-9378(91)91440-8

[64] Holborn MA, Ford G, Turner S, et al. The NESHIE and CP Genetics Resource (NCGR): a database of genes and variants reported in neonatal encephalopathy with suspected hypoxic ischemic encephalopathy (NESHIE) and consequential cerebral palsy (CP). Genomics. 2022;114(6):110508. 10.1016/j.ygeno.2022.110508. 36270382 10.1016/j.ygeno.2022.110508 PMC9726645

[65] Caramelo I, Coelho M, Rosado M, et al. Biomarkers of hypoxic–ischemic encephalopathy: a systematic review. World J Pediatr. 2023;19(6):505–48. 10.1007/s12519-023-00698-7. 37084165 10.1007/s12519-023-00698-7 PMC10199106

[66] Sánchez-Illana Á, Piñeiro-Ramos JD, Kuligowski J. Small molecule biomarkers for neonatal hypoxic ischemic encephalopathy. Semin Fetal Neonatal Med. 2020;25(2):101084. 10.1016/j.siny.2020.101084. 31983670 10.1016/j.siny.2020.101084

[67] Efstathiou N, Theodoridis G, Sarafidis K. Understanding neonatal hypoxic-ischemic encephalopathy with metabolomics. Hippokratia. 2017;21(3):115–23. 30479472 PMC6248003

[68] Montaldo P, Burgod C, Herberg JA, et al. Whole-blood gene expression profile after hypoxic-ischemic encephalopathy. JAMA. 2024;7(2):e2354433-e. 10.1001/jamanetworkopen.2023.54433. 10.1001/jamanetworkopen.2023.54433 PMC10837749 38306098

[69] Waddington CH. The epigenotype 1942. Int J Epidemiol. 2012;41(1):10–3. 10.1093/ije/dyr184. 22186258 10.1093/ije/dyr184

[70] Waddington C. The strategy of the genes. A discussion of some aspects of theoretical biology. London: George Allen & Unwin; 1957.

[71] Wu H, Sun YE. Epigenetic regulation of stem cell differentiation. Pediatr Res. 2006;59(4):21–5. 10.1203/01.pdr.0000203565.76028.2a. 16549544 10.1203/01.pdr.0000203565.76028.2a

[72] Basu A, Tiwari VK. Epigenetic reprogramming of cell identity: lessons from development for regenerative medicine. Clin Epigenet. 2021;13(1):144. 10.1186/s13148-021-01131-4. 10.1186/s13148-021-01131-4 PMC8305869 34301318

[73] Wu Y-L, Lin Z-J, Li C-C, et al. Epigenetic regulation in metabolic diseases: mechanisms and advances in clinical study. Sig Transduct Target Ther. 2023;8(1):98. 10.1038/s41392-023-01333-7. 10.1038/s41392-023-01333-7 PMC9981733 36864020

[74] Moore LD, Le T, Fan G. DNA methylation and its basic function. Neuropsychopharmacol. 2013;38(1):23–38. 10.1038/npp.2012.112. 10.1038/npp.2012.112 PMC3521964 22781841

[75] Lyko F. The DNA methyltransferase family: a versatile toolkit for epigenetic regulation. Nat Rev Genet. 2018;19(2):81–92. 10.1038/nrg.2017.80. 29033456 10.1038/nrg.2017.80

[76] Deaton AM, Bird A. Cpg islands and the regulation of transcription. Genes Dev. 2011;25(10):1010–22. 10.1101/gad.2037511. 21576262 10.1101/gad.2037511 PMC3093116

[77] Miranda TB, Jones PA. DNA methylation: the nuts and bolts of repression. J Cell Physiol. 2007;213(2):384–90. 10.1002/jcp.21224. 17708532 10.1002/jcp.21224

[78] Parle-Mcdermott A, Harrison A. DNA methylation: a timeline of methods and applications. Front Genet. 2011;2:74. 10.3389/fgene.2011.00074. 22303369 10.3389/fgene.2011.00074 PMC3268627

[79] Li Y, Tollefsbol TO. DNA methylation detection: bisulfite genomic sequencing analysis. Methods Mol Biol. 2011;791:11–21. 10.1007/978-1-61779-316-5_2. 21913068 10.1007/978-1-61779-316-5_2 PMC3233226

[80] Handy DE, Castro R, Loscalzo J. Epigenetic modifications: basic mechanisms and role in cardiovascular disease. Circulation. 2011;123(19):2145–56. 10.1161/circulationaha.110.956839. 21576679 10.1161/CIRCULATIONAHA.110.956839 PMC3107542

[81] Mariño-Ramírez L, Kann MG, Shoemaker BA, et al. Histone structure and nucleosome stability. Expert Rev Proteomics. 2005;2(5):719–29. 10.1586/14789450.2.5.719. 16209651 10.1586/14789450.2.5.719 PMC1831843

[82] Zhao Y, Garcia BA. Comprehensive catalog of currently documented histone modifications. Cold Spring Harb Perspect Biol. 2015;7(9):a025064. 10.1101/cshperspect.a025064. 26330523 10.1101/cshperspect.a025064 PMC4563710

[83] Klemm SL, Shipony Z, Greenleaf WJ. Chromatin accessibility and the regulatory epigenome. Nat Rev Genet. 2019;20(4):207–20. 10.1038/s41576-018-0089-8. 30675018 10.1038/s41576-018-0089-8

[84] Grandi FC, Modi H, Kampman L, et al. Chromatin accessibility profiling by ATAC-seq. Nat Protoc. 2022;17(6):1518–52. 10.1038/s41596-022-00692-9. 35478247 10.1038/s41596-022-00692-9 PMC9189070

[85] Iyer MK, Niknafs YS, Malik R, et al. The landscape of long noncoding RNAs in the human transcriptome. Nat Genet. 2015;47(3):199–208. 10.1038/ng.3192. 25599403 10.1038/ng.3192 PMC4417758

[86] Kaikkonen MU, Lam MT, Glass CK. Non-coding RNAs as regulators of gene expression and epigenetics. Cardiovasc Res. 2011;90(3):430–40. 10.1093/cvr/cvr097. 21558279 10.1093/cvr/cvr097 PMC3096308

[87] Wei J-W, Huang K, Yang C, et al. Non-coding RNAs as regulators in epigenetics (review). Oncol Rep. 2017;37(1):3–9. 10.3892/or.2016.5236. 27841002 10.3892/or.2016.5236

[88] Wang M, Yu F, Wu W, et al. Circular RNAs: a novel type of non-coding RNA and their potential implications in antiviral immunity. Int J Biol Sci. 2017;13(12):1497–506. 10.7150/ijbs.22531. 29230098 10.7150/ijbs.22531 PMC5723916

[89] Arnold PR, Wells AD, Li XC. Diversity and emerging roles of enhancer RNA in regulation of gene expression and cell fate. Front Cell Dev Biol. 2020. 10.3389/fcell.2019.00377. 10.3389/fcell.2019.00377 PMC6971116 31993419

[90] Johanson TM, Lew AM, Chong MM. Microrna-independent roles of the RNase III enzymes Drosha and Dicer. Open Biol. 2013;3(10):130144. 10.1098/rsob.130144. 24153005 10.1098/rsob.130144 PMC3814725

[91] Mattick JS, Amaral PP, Carninci P, et al. Long non-coding RNAs: definitions, functions, challenges and recommendations. Nat Rev Mol Cell Biol. 2023;24(6):430–47. 10.1038/s41580-022-00566-8. 36596869 10.1038/s41580-022-00566-8 PMC10213152

[92] Statello L, Guo C-J, Chen L-L, et al. Gene regulation by long non-coding RNAs and its biological functions. Nat Rev Mol Cell Biol. 2021;22(2):96–118. 10.1038/s41580-020-00315-9. 33353982 10.1038/s41580-020-00315-9 PMC7754182

[93] Mangiavacchi A, Morelli G, Orlando V. Behind the scenes: how RNA orchestrates the epigenetic regulation of gene expression. Front Cell Dev Biol. 2023. 10.3389/fcell.2023.1123975. 10.3389/fcell.2023.1123975 PMC9905133 36760365

[94] D’Urso A, Brickner JH. Mechanisms of epigenetic memory. Trends Genet. 2014;30(6):230–6. 10.1016/j.tig.2014.04.004. 24780085 10.1016/j.tig.2014.04.004 PMC4072033

[95] Skinner MK. Environmental epigenetic transgenerational inheritance and somatic epigenetic mitotic stability. Epigenetics. 2011;6(7):838–42. 10.4161/epi.6.7.16537. 21637037 10.4161/epi.6.7.16537 PMC5703187

[96] Fitz-James MH, Cavalli G. Molecular mechanisms of transgenerational epigenetic inheritance. Nat Rev Genet. 2022;23(6):325–41. 10.1038/s41576-021-00438-5. 34983971 10.1038/s41576-021-00438-5 PMC7619059

[97] Messerschmidt DM, Knowles BB, Solter D. DNA methylation dynamics during epigenetic reprogramming in the germline and preimplantation embryos. Genes Dev. 2014;28(8):812–28. 10.1101/gad.234294.113. 24736841 10.1101/gad.234294.113 PMC4003274

[98] Wenger A, Biran A, Alcaraz N, et al. Symmetric inheritance of parental histones governs epigenome maintenance and embryonic stem cell identity. Nat Genet. 2023;55(9):1567–78. 10.1038/s41588-023-01476-x. 37666988 10.1038/s41588-023-01476-x PMC10484787

[99] Budhavarapu VN, Chavez M, Tyler JK. How is epigenetic information maintained through DNA replication? Epigenetics Chromatin. 2013;6(1):32. 10.1186/1756-8935-6-32. 24225278 10.1186/1756-8935-6-32 PMC3852060

[100] Ohkura H. Meiosis: an overview of key differences from mitosis. Cold Spring Harb Perspect Biol. 2015. 10.1101/cshperspect.a015859. 10.1101/cshperspect.a015859 PMC4448623 25605710

[101] Larose H, Shami AN, Abbott H, et al. Gametogenesis: A journey from inception to conception. Curr Top Dev Biol. 2019;132:257–310. 10.1016/bs.ctdb.2018.12.006. 30797511 10.1016/bs.ctdb.2018.12.006 PMC7133493

[102] Ramakrishna NB, Murison K, Miska EA, et al. Epigenetic regulation during primordial germ cell development and differentiation. Sex Dev. 2021;15(5–6):411–31. 10.1159/000520412. 34847550 10.1159/000520412

[103] Hajkova P. Epigenetic reprogramming in the germline: towards the ground state of the epigenome. Philos Trans R Soc Lond B Biol Sci. 2011;366(1575):2266–73. 10.1098/rstb.2011.0042. 21727132 10.1098/rstb.2011.0042 PMC3130423

[104] Kota SK, Feil R. Epigenetic transitions in germ cell development and meiosis. Dev Cell. 2010;19(5):675–86. 10.1016/j.devcel.2010.10.009. 21074718 10.1016/j.devcel.2010.10.009

[105] Ben Maamar M, Nilsson EE, Skinner MK. Epigenetic transgenerational inheritance, gametogenesis and germline development. Biol Reprod. 2021;105(3):570–92. 10.1093/biolre/ioab085. 33929020 10.1093/biolre/ioab085 PMC8444706

[106] Pomerening JR. Protein Kinase Assay. In: Dubitzky W, Wolkenhauer O, Cho K-H, Yokota H, editors. Encyclopedia of Systems Biology. NY: Springer, New York New York; 2013.

[107] Akmal M, Aa A, Widodo MA, et al. The important role of protamine in spermatogenesis and quality of sperm: A mini review. Asian Pac J Reprod. 2016;5(5):357–60. 10.1016/j.apjr.2016.07.013.

[108] Tóth KF, Pezic D, Stuwe E, et al. The piRNA pathway guards the germline genome against transposable elements. Adv Exp Med Biol. 2016;886:51–77. 10.1007/978-94-017-7417-8_4. 26659487 10.1007/978-94-017-7417-8_4 PMC4991928

[109] Zhang Y, Sirard MA. Epigenetic inheritance of acquired traits through DNA methylation. Anim Front. 2021;11(6):19–27. 10.1093/af/vfab052. 10.1093/af/vfab052 PMC8683130 34934526

[110] Nilsson EE, Sadler-Riggleman I, Skinner MK. Environmentally induced epigenetic transgenerational inheritance of disease. Environ Epigenet. 2018;4(2):dvy016. 10.1093/eep/dvy016. 30038800 10.1093/eep/dvy016 PMC6051467

[111] O’Neill C. The epigenetics of embryo development. Anim Front. 2015;5(1):42–9. 10.2527/af.2015-0007.

[112] Breton-Larrivée M, Elder E, McGraw S. DNA methylation, environmental exposures and early embryo development. Anim Reprod. 2019;16(3):465–74. 10.21451/1984-3143-ar2019-0062. 32435290 10.21451/1984-3143-AR2019-0062 PMC7234019

[113] Jones MJ, Goodman SJ, Kobor MS. DNA methylation and healthy human aging. Aging Cell. 2015;14(6):924–32. 10.1111/acel.12349. 25913071 10.1111/acel.12349 PMC4693469

[114] Roseboom TJ, Painter RC, van Abeelen AFM, et al. Hungry in the womb: what are the consequences? Lessons from the Dutch famine. Maturitas. 2011;70(2):141–5. 10.1016/j.maturitas.2011.06.017. 21802226 10.1016/j.maturitas.2011.06.017

[115] Heijmans BT, Tobi EW, Stein AD, et al. Persistent epigenetic differences associated with prenatal exposure to famine in humans. Proc Natl Acad Sci U S A. 2008;105(44):17046–9. 10.1073/pnas.0806560105. 18955703 10.1073/pnas.0806560105 PMC2579375

[116] Stein AD, Pierik FH, Verrips GH, et al. Maternal exposure to the Dutch famine before conception and during pregnancy: quality of life and depressive symptoms in adult offspring. Epidemiology. 2009;20(6):909–15. 10.1097/EDE.0b013e3181b5f227. 19752733 10.1097/EDE.0b013e3181b5f227 PMC3850290

[117] Horsthemke B. A critical view on transgenerational epigenetic inheritance in humans. Nat Commun. 2018;9(1):2973. 10.1038/s41467-018-05445-5. 30061690 10.1038/s41467-018-05445-5 PMC6065375

[118] Macdonald WA. Epigenetic mechanisms of genomic imprinting: common themes in the regulation of imprinted regions in mammals, plants, and insects. Genet Res Int. 2012;2012:585024. 10.1155/2012/585024. 22567394 10.1155/2012/585024 PMC3335465

[119] Regmi S, Giha L, Ali A, et al. Methylation is maintained specifically at imprinting control regions but not other DMRs associated with imprinted genes in mice bearing a mutation in the Dnmt1 intrinsically disordered domain. Front Cell Dev Biol. 2023. 10.3389/fcell.2023.1192789. 10.3389/fcell.2023.1192789 PMC10436486 37601113

[120] Kwon EJ, Kim YJ. What is fetal programming? A lifetime health is under the control of in utero health. Obstet Gynecol Sci. 2017;60(6):506–19. 10.5468/ogs.2017.60.6.506. 29184858 10.5468/ogs.2017.60.6.506 PMC5694724

[121] Barker DJ, Bull AR, Osmond C, et al. Fetal and placental size and risk of hypertension in adult life. BMJ. 1990;301(6746):259–62. 10.1136/bmj.301.6746.259. 2390618 10.1136/bmj.301.6746.259 PMC1663477

[122] Saffery R, Novakovic B. Epigenetics as the mediator of fetal programming of adult onset disease: what is the evidence? Acta Obstet Gynecol Scand. 2014;93(11):1090–8. 10.1111/aogs.12431. 24835110 10.1111/aogs.12431

[123] Lacagnina S. The developmental origins of health and disease (DOHaD). Am J Lifestyle Med. 2020;14(1):47–50. 10.1177/1559827619879694. 31903081 10.1177/1559827619879694 PMC6933571

[124] Zhu Z, Cao F, Li X. Epigenetic programming and fetal metabolic programming. Front Endocrinol. 2019. 10.3389/fendo.2019.00764. 10.3389/fendo.2019.00764 PMC6901800 31849831

[125] Stevenson K, Lillycrop KA, Silver MJ. Fetal programming and epigenetics. Curr Opin Endocrinol Diabetes Obes. 2020;13:1–6. 10.1016/j.coemr.2020.07.005.

[126] Suresh MV, Balijepalli S, Solanki S, et al. Hypoxia-inducible factor 1α and its role in lung injury: adaptive or maladaptive. Inflammation. 2023;46(2):491–508. 10.1007/s10753-022-01769-z. 36596930 10.1007/s10753-022-01769-z PMC9811056

[127] Schlegel C, Liu K, Spring B, et al. Decreased expression of hypoxia-inducible factor 1α (HIF-1α) in cord blood monocytes under anoxia. Pediatr Res. 2023;93(4):870–7. 10.1038/s41390-022-02193-7. 35906309 10.1038/s41390-022-02193-7 PMC10033401

[128] Ziello JE, Jovin IS, Huang Y. Hypoxia-inducible factor (HIF)-1 regulatory pathway and its potential for therapeutic intervention in malignancy and ischemia. Yale J Biol Med. 2007;80(2):51–60. 18160990 PMC2140184

[129] Masoud GN, Li W. HIF-1α pathway: role, regulation and intervention for cancer therapy. Acta Pharm Sin B. 2015;5(5):378–89. 10.1016/j.apsb.2015.05.007. 26579469 10.1016/j.apsb.2015.05.007 PMC4629436

[130] Yfantis A, Mylonis I, Chachami G, et al. Transcriptional response to hypoxia: the role of HIF-1-associated co-regulators. Cells. 2023;12(5):798. 10.3390/cells12050798. 36899934 10.3390/cells12050798 PMC10001186

[131] Majmundar AJ, Wong WJ, Simon MC. Hypoxia-inducible factors and the response to hypoxic stress. Mol Cell. 2010;40(2):294–309. 10.1016/j.molcel.2010.09.022. 20965423 10.1016/j.molcel.2010.09.022 PMC3143508

[132] Weidemann A, Johnson RS. Biology of HIF-1α. Cell Death Differ. 2008;15(4):621–7. 10.1038/cdd.2008.12. 18259201 10.1038/cdd.2008.12

[133] Zhang M, Liu Q, Meng H, et al. Ischemia-reperfusion injury: molecular mechanisms and therapeutic targets. Sig Transduct Target Ther. 2024;9(1):12. 10.1038/s41392-023-01688-x. 10.1038/s41392-023-01688-x PMC10772178 38185705

[134] Liang X, Liu X, Lu F, et al. HIF1α signaling in the endogenous protective responses after neonatal brain hypoxia-ischemia. Dev Neurosci. 2019. 10.1159/000495879. 10.1159/000495879 PMC6728223 30836371

[135] Berchner-Pfannschmidt U, Frede S, Wotzlaw C, et al. Imaging of the hypoxia-inducible factor pathway: insights into oxygen sensing. Eur Respir J. 2008;32(1):210. 10.1183/09031936.00013408. 18591338 10.1183/09031936.00013408

[136] Kukec E, Goričar K, Dolžan V, et al. HIF1A polymorphisms do not modify the risk of epilepsy nor cerebral palsy after neonatal hypoxic-ischemic encephalopathy. Brain Res. 2021;1757:147281. 10.1016/j.brainres.2021.147281. 33515534 10.1016/j.brainres.2021.147281

[137] Filippi L, Scaramuzzo RT, Pascarella F, et al. Fetal oxygenation in the last weeks of pregnancy evaluated through the umbilical cord blood gas analysis. Front Pediatr. 2023;11:1140021. 10.3389/fped.2023.1140021. 37152310 10.3389/fped.2023.1140021 PMC10160648

[138] Engelhardt S, Al-Ahmad AJ, Gassmann M, et al. Hypoxia selectively disrupts brain microvascular endothelial tight junction complexes through a hypoxia-inducible factor-1 (HIF-1) dependent mechanism. J Cell Physiol. 2014;229(8):1096–105. 10.1002/jcp.24544. 24375098 10.1002/jcp.24544

[139] Amin N, Chen S, Ren Q, et al. Hypoxia Inducible Factor-1α attenuates ischemic brain damage by modulating inflammatory response and glial activity. Cells. 2021;10(6):1359. 10.3390/cells10061359. 34205911 10.3390/cells10061359 PMC8229365

[140] Hölscher M, Schäfer K, Krull S, et al. Unfavourable consequences of chronic cardiac HIF-1α stabilization. Cardiovasc Res. 2012;94(1):77–86. 10.1093/cvr/cvs014. 22258630 10.1093/cvr/cvs014

[141] Zhang Z, Yao L, Yang J, et al. PI3K/Akt and HIF-1 signaling pathway in hypoxia-ischemia (Review). Mol Med Rep. 2018;18(4):3547–54. 10.3892/mmr.2018.9375. 30106145 10.3892/mmr.2018.9375 PMC6131612

[142] Li C, Xiong W, Liu X, et al. Hypomethylation at non-CpG/CpG sites in the promoter of HIF-1α gene combined with enhanced H3K9Ac modification contribute to maintain higher HIF-1α expression in breast cancer. Oncogenesis. 2019;8(4):26. 10.1038/s41389-019-0135-1. 30940798 10.1038/s41389-019-0135-1 PMC6445832

[143] Nam HJ, Baek SH. Epigenetic regulation of the hypoxic response. CRPHYS. 2019;7:1–8. 10.1016/j.cophys.2018.11.007.

[144] Wenger RH, Kvietikova I, Rolfs A, et al. Oxygen-regulated erythropoietin gene expression is dependent on a CpG methylation-free hypoxia-inducible factor-1 DNA-binding site. Eur J Biochem. 1998;253(3):771–7. 10.1046/j.1432-1327.1998.2530771.x. 9654078 10.1046/j.1432-1327.1998.2530771.x

[145] Kumral A, Tuzun F, Tugyan K, et al. Role of epigenetic regulatory mechanisms in neonatal hypoxic-ischemic brain injury. Early Hum Dev. 2013;89(3):165–73. 10.1016/j.earlhumdev.2012.09.016. 23046993 10.1016/j.earlhumdev.2012.09.016

[146] Kim SH, Jeong JW, Park JA, et al. Regulation of the HIF-1alpha stability by histone deacetylases. Oncol Rep. 2007;17(3):647–51. 17273746

[147] Beyer S, Kristensen MM, Jensen KS, et al. The histone demethylases JMJD1A and JMJD2B are transcriptional targets of hypoxia-inducible factor HIF. J Biol Chem. 2008;283(52):36542–52. 10.1074/jbc.M804578200. 18984585 10.1074/jbc.M804578200 PMC2662309

[148] Li T, Mao C, Wang X, et al. Epigenetic crosstalk between hypoxia and tumor driven by HIF regulation. J Exp Clin Cancer Res. 2020;39(1):224. 10.1186/s13046-020-01733-5. 33109235 10.1186/s13046-020-01733-5 PMC7592369

[149] Taguchi A, Yanagisawa K, Tanaka M, et al. Identification of hypoxia-inducible factor-1 alpha as a novel target for miR-17-92 microRNA cluster. Cancer Res. 2008;68(14):5540–5. 10.1158/0008-5472.Can-07-6460. 18632605 10.1158/0008-5472.CAN-07-6460

[150] Rane S, He M, Sayed D, et al. Downregulation of miR-199a derepresses hypoxia-inducible factor-1alpha and Sirtuin 1 and recapitulates hypoxia preconditioning in cardiac myocytes. Circ Res. 2009;104(7):879–86. 10.1161/circresaha.108.193102. 19265035 10.1161/CIRCRESAHA.108.193102 PMC3332328

[151] Lei Z, Li B, Yang Z, et al. Regulation of HIF-1alpha and VEGF by miR-20b tunes tumor cells to adapt to the alteration of oxygen concentration. PLoS ONE. 2009;4(10):e7629. 10.1371/journal.pone.0007629. 19893619 10.1371/journal.pone.0007629 PMC2764090

[152] Kulshreshtha R, Ferracin M, Wojcik SE, et al. A microRNA signature of hypoxia. Mol Cell Biol. 2007;27(5):1859–67. 10.1128/mcb.01395-06. 17194750 10.1128/MCB.01395-06 PMC1820461

[153] Ivan M, Huang X. miR-210: fine-tuning the hypoxic response. Adv Exp Med Biol. 2014;772:205–27. 10.1007/978-1-4614-5915-6_10. 24272361 10.1007/978-1-4614-5915-6_10 PMC4515752

[154] Hui X, Al-Ward H, Shaher F, et al. The role of miR-210 in the biological system: a current overview. Hum Hered. 2020;84(6):233–9. 10.1159/000509280. 10.1159/000509280 32906127

[155] Whitehead CL, Teh WT, Walker SP, et al. Circulating MicroRNAs in maternal blood as potential biomarkers for fetal hypoxia in-utero. PLoS ONE. 2013;8(11):e78487. 10.1371/journal.pone.0078487. 24282500 10.1371/journal.pone.0078487 PMC3839903

[156] Looney A-M, Walsh BH, Moloney G, et al. Downregulation of umbilical cord blood levels of miR-374a in neonatal hypoxic ischemic encephalopathy. J Pediatr. 2015;167(2):269-73.e2. 10.1016/j.jpeds.2015.04.060. 26001314 10.1016/j.jpeds.2015.04.060

[157] Looney AM, O’Sullivan MP, Ahearne CE, et al. Altered expression of umbilical cord blood levels of miR-181b and its downstream target mUCH-L1 in infants with moderate and severe neonatal hypoxic-schaemic encephalopathy. Mol Neurobiol. 2019;56(5):3657–63. 10.1007/s12035-018-1321-4. 30178296 10.1007/s12035-018-1321-4

[158] O’Sullivan MP, Looney AM, Moloney GM, et al. Validation of altered umbilical cord blood microRNA expression in neonatal hypoxic-ischemic encephalopathy. JAMA Neurol. 2019;76(3):333–41. 10.1001/jamaneurol.2018.4182. 30592487 10.1001/jamaneurol.2018.4182 PMC6439719

[159] Wang Z, Liu Y, Shao M, et al. Combined prediction of miR-210 and miR-374a for severity and prognosis of hypoxic-ischemic encephalopathy. Brain Behav. 2018;8(1):e00835. 10.1002/brb3.835. 29568675 10.1002/brb3.835 PMC5853646

[160] Penas C, Navarro X. Epigenetic modifications associated to neuroinflammation and neuropathic pain after neural trauma. Front Cell Neurosci. 2018;12:158. 10.3389/fncel.2018.00158. 29930500 10.3389/fncel.2018.00158 PMC5999732

[161] Tan SYX, Zhang J, Tee WW. Epigenetic regulation of inflammatory signaling and inflammation-induced cancer. Front Cell Dev Biol. 2022;10:931493. 10.3389/fcell.2022.931493. 35757000 10.3389/fcell.2022.931493 PMC9213816

[162] Nanduri J, Makarenko V, Reddy VD, et al. Epigenetic regulation of hypoxic sensing disrupts cardiorespiratory homeostasis. PNAS. 2012;109(7):2515–20. 10.1073/pnas.1120600109. 22232674 10.1073/pnas.1120600109 PMC3289330

[163] Sato K, Kumagai N, Suzuki N. Alteration of the DNA methylation signature of renal erythropoietin-producing cells governs the sensitivity to drugs targeting the hypoxia-response pathway in kidney disease progression. Front Genet. 2019;10:1134. 10.3389/fgene.2019.01134. 31798631 10.3389/fgene.2019.01134 PMC6863978

[164] Iwamura Y, Nakai T, Kato K, et al. Erythropoietin production in embryonic neural cells is controlled by hypoxia-inducible factors and histone deacetylases in an undifferentiated state. bioRxiv. 2024:2024.02.28.582479. 10.1101/2024.02.28.582479

[165] Haase VH. Regulation of erythropoiesis by hypoxia-inducible factors. Blood Rev. 2013;27(1):41–53. 10.1016/j.blre.2012.12.003. 23291219 10.1016/j.blre.2012.12.003 PMC3731139

[166] Kim J, Lee H, Yi SJ, et al. Gene regulation by histone-modifying enzymes under hypoxic conditions: a focus on histone methylation and acetylation. Exp Mol Med. 2022;54(7):878–89. 10.1038/s12276-022-00812-1. 35869366 10.1038/s12276-022-00812-1 PMC9355978

[167] Li P, Wang Y, Sun Y, et al. N6-methyladenosine RNA methylation: From regulatory mechanisms to potential clinical applications. Front Cell Dev Biol. 2022;10:1055808. 10.3389/fcell.2022.1055808. 36407103 10.3389/fcell.2022.1055808 PMC9669580

[168] Yue Y, Liu J, He C. RNA N6-methyladenosine methylation in post-transcriptional gene expression regulation. Genes Dev. 2015;29(13):1343–55. 10.1101/gad.262766.115. 26159994 10.1101/gad.262766.115 PMC4511210

[169] Shi H, Wei J, He C. Where, when, and how: context-dependent functions of RNA methylation writers, readers, and erasers. Mol Cell. 2019;74(4):640–50. 10.1016/j.molcel.2019.04.025. 31100245 10.1016/j.molcel.2019.04.025 PMC6527355

[170] Zou J, Liu H, Tan W, et al. Dynamic regulation and key roles of ribonucleic acid methylation. Front Cell Neurosci. 2022;16:158083. 10.3389/fncel.2022.1058083. 10.3389/fncel.2022.1058083 PMC9806184 36601431

[171] Knopik VS, Marceau K, Bidwell LC, et al. Prenatal substance exposure and offspring development: does DNA methylation play a role? Neurotoxicol Teratol. 2019;71:50–63. 10.1016/j.ntt.2018.01.009. 29408446 10.1016/j.ntt.2018.01.009 PMC6093803

[172] Banik A, Kandilya D, Ramya S, et al. Maternal factors that induce epigenetic changes contribute to neurological disorders in offspring. Genes (Basel). 2017;8(6):150. 10.3390/genes8060150. 28538662 10.3390/genes8060150 PMC5485514

[173] Smith TF, Schmidt-Kastner R, McGeary JE, et al. Pre- and perinatal ischemia-hypoxia, the ischemia-hypoxia response pathway, and ADHD risk. Behav Genet. 2016;46(3):467–77. 10.1007/s10519-016-9784-4. 26920003 10.1007/s10519-016-9784-4

[174] Dumont U, Sanchez S, Olivier B, et al. Maternal alcoholism and neonatal hypoxia-ischemia: Neuroprotection by stilbenoid polyphenols. Brain Res. 2020;1738:146798. 10.1016/j.brainres.2020.146798. 32229200 10.1016/j.brainres.2020.146798

[175] Haycock PC. Fetal alcohol spectrum disorders: the epigenetic perspective. Biol Reprod. 2009;81(4):607–17. 10.1095/biolreprod.108.074690. 19458312 10.1095/biolreprod.108.074690

[176] Mead EA, Sarkar DK. Fetal alcohol spectrum disorders and their transmission through genetic and epigenetic mechanisms. Front Genet. 2014;5:154. 10.3389/fgene.2014.00154. 24917878 10.3389/fgene.2014.00154 PMC4040491

[177] Alberry B, Laufer BI, Chater-Diehl E, et al. Epigenetic impacts of early life stress in fetal alcohol spectrum disorders shape the neurodevelopmental continuum. Front Mol Neurosci. 2021;14:671891. 10.3389/fnmol.2021.671891. 34149355 10.3389/fnmol.2021.671891 PMC8209299

[178] Olivier L, Curfs LM, Viljoen DL. Fetal alcohol spectrum disorders: Prevalence rates in South Africa. S Afr Med J. 2016;106(6 Suppl 1):S103–6. 10.7196/SAMJ.2016.v106i6.11009. 27245541 10.7196/SAMJ.2016.v106i6.11009

[179] Maccani JZ, Maccani MA. Altered placental DNA methylation patterns associated with maternal smoking: current perspectives. Adv Genomics Genet. 2015;2015(5):205–14. 10.2147/agg.S61518. 26203295 10.2147/AGG.S61518 PMC4507353

[180] Suter M, Ma J, Harris A, et al. Maternal tobacco use modestly alters correlated epigenome-wide placental DNA methylation and gene expression. Epigenetics. 2011;6(11):1284–94. 10.4161/epi.6.11.17819. 21937876 10.4161/epi.6.11.17819 PMC3242811

[181] Ivorra C, Fraga MF, Bayón GF, et al. DNA methylation patterns in newborns exposed to tobacco in utero. J Transl Med. 2015;13:25. 10.1186/s12967-015-0384-5. 25623364 10.1186/s12967-015-0384-5 PMC4312439

[182] Maddox TR, Haas J, Andrews L, et al. Abnormal presentation of hypoxic ischemic encephalopathy attributed to polysubstance exposure. Am J Case Rep. 2019;20:1715–8. 10.12659/ajcr.918091. 31747388 10.12659/AJCR.918091 PMC6878964

[183] Kumar M, Saadaoui M, Al KS. Infections and pregnancy: effects on maternal and child health. Front Cell Infect Microbiol. 2022;12:873253. 10.3389/fcimb.2022.873253. 35755838 10.3389/fcimb.2022.873253 PMC9217740

[184] Abu-Raya B, Michalski C, Sadarangani M, et al. Maternal immunological adaptation during normal pregnancy. Front Immunol. 2020;11:575197. 10.3389/fimmu.2020.575197. 33133091 10.3389/fimmu.2020.575197 PMC7579415

[185] Cohen R, Gutvirtz G, Wainstock T, et al. Maternal urinary tract infection during pregnancy and long-term infectious morbidity of the offspring. Early Hum Dev. 2019;136:54–9. 10.1016/j.earlhumdev.2019.07.002. 31319353 10.1016/j.earlhumdev.2019.07.002

[186] Balachandran L, Jacob L, Al Awadhi R, et al. Urinary tract infection in pregnancy and its effects on maternal and perinatal outcome: a retrospective study. Cureus. 2022;14(1):e21500. 10.7759/cureus.21500. 35223276 10.7759/cureus.21500 PMC8860729

[187] Collier SA, Rasmussen SA, Feldkamp ML, et al. Prevalence of self-reported infection during pregnancy among control mothers in the National Birth Defects Prevention Study. Birth Defects Res A Clin Mol Terato. 2009;85(3):193–201. 10.1002/bdra.20540. 10.1002/bdra.20540 19086018

[188] Gigi RMS, Buitrago-Garcia D, Taghavi K, et al. Vulvovaginal yeast infections during pregnancy and perinatal outcomes: systematic review and meta-analysis. BMC Womens Health. 2023;23(1):116. 10.1186/s12905-023-02258-7. 36944953 10.1186/s12905-023-02258-7 PMC10029297

[189] Dorothy Chiwoniso N, Andrew M-M, Remco PHP, et al. Prevalence, incidence and associated risk factors of STIs during pregnancy in South Africa. Sex Transm Infect. 2021;97(5):375. 10.1136/sextrans-2020-054631. 33004610 10.1136/sextrans-2020-054631 PMC8012394

[190] Mullins TLK, Li SX, Bethel J, et al. Sexually transmitted infections and immune activation among HIV-infected but virally suppressed youth on antiretroviral therapy. J Clin Virol. 2018;102:7–11. 10.1016/j.jcv.2018.02.001. 29454196 10.1016/j.jcv.2018.02.001 PMC5889960

[191] Basil P, Li Q, Dempster EL, et al. Prenatal maternal immune activation causes epigenetic differences in adolescent mouse brain. Transl Psychiatry. 2014;4(9):e434. 10.1038/tp.2014.80. 25180573 10.1038/tp.2014.80 PMC4203009

[192] Bobdiwala S, Loudon J. Hypoxic ischaemic encephalopathy and infection, is there an association? Arch Dis Child Fetal Neonatal Ed. 2012;97(Suppl 1):A90. 10.1136/fetalneonatal-2012-301809.292.

[193] Yang W, Wang L, Tian T, et al. Maternal hypertensive disorders in pregnancy and risk of hypoxic-ischemia encephalopathy. J Matern Fetal Neonatal Med. 2021;34(11):1754–62. 10.1080/14767058.2019.1647529. 31331218 10.1080/14767058.2019.1647529

[194] Pamungkas S, Irwinda R, Wibowo N. High morbidity of preterm neonates in pregnancy with preeclampsia: a retrospective study in indonesia. JSAFOG. 2022. 10.5005/jp-journals-10006-2023.

[195] Knihtilä HM, Kachroo P, Shadid I, et al. Cord blood DNA methylation signatures associated with preeclampsia are enriched for cardiovascular pathways: insights from the VDAART trial. EBioMedicine. 2023;98:104890. 10.1016/j.ebiom.2023.104890. 37995466 10.1016/j.ebiom.2023.104890 PMC10709000

[196] Cirkovic A, Garovic V, Milin Lazovic J, et al. Systematic review supports the role of DNA methylation in the pathophysiology of preeclampsia: a call for analytical and methodological standardization. Biol Sex Differ. 2020;11(1):36. 10.1186/s13293-020-00313-8. 32631423 10.1186/s13293-020-00313-8 PMC7336649

[197] Ashraf UM, Hall DL, Rawls AZ, et al. Epigenetic processes during preeclampsia and effects on fetal development and chronic health. Clin Sci (Lond). 2021;135(19):2307–27. 10.1042/cs20190070. 34643675 10.1042/CS20190070 PMC8948502

[198] Nomura Y, Lambertini L, Rialdi A, et al. Global methylation in the placenta and umbilical cord blood from pregnancies with maternal gestational diabetes, preeclampsia, and obesity. Reprod Sci. 2014;21(1):131–7. 10.1177/1933719113492206. 23765376 10.1177/1933719113492206 PMC3857768

[199] Hayashi M, Sakata M, Takeda T, et al. Induction of glucose transporter 1 expression through hypoxia-inducible factor 1alpha under hypoxic conditions in trophoblast-derived cells. J Endocrinol. 2004;183(1):145–54. 10.1677/joe.1.05599. 15525582 10.1677/joe.1.05599

[200] Wang C, Liu W, Liu Z, et al. Hypoxia inhibits myogenic differentiation through p53 protein-dependent induction of Bhlhe40 protein. J Biol Chem. 2015;290(50):29707–16. 10.1074/jbc.M115.688671. 26468276 10.1074/jbc.M115.688671 PMC4706003

[201] Dias S, Willmer T, Adam S, et al. The role of maternal DNA methylation in pregnancies complicated by gestational diabetes. Front Clin Diabetes Healthc. 2022;3:982665. 10.3389/fcdhc.2022.982665. 36992770 10.3389/fcdhc.2022.982665 PMC10012132

[202] Haertle L, El Hajj N, Dittrich M, et al. Epigenetic signatures of gestational diabetes mellitus on cord blood methylation. Clin Epigenetics. 2017;9:28. 10.1186/s13148-017-0329-3. 28360945 10.1186/s13148-017-0329-3 PMC5368916

[203] Awamleh Z, Butcher DT, Hanley A, et al. Exposure to gestational diabetes mellitus (GDM) alters DNA methylation in placenta and fetal cord blood. Diabetes Res Clin Pract. 2021;174:108690. 10.1016/j.diabres.2021.108690. 33549677 10.1016/j.diabres.2021.108690

[204] Dłuski DF, Wolińska E, Skrzypczak M. Epigenetic changes in gestational diabetes mellitus. Int J Mol Sci. 2021;22(14):7649. 10.3390/ijms22147649. 34299269 10.3390/ijms22147649 PMC8303885

[205] Yang SL, Wu C, Xiong ZF, et al. Progress on hypoxia-inducible factor-3: Its structure, gene regulation and biological function (Review). Mol Med Rep. 2015;12(2):2411–6. 10.3892/mmr.2015.3689. 25936862 10.3892/mmr.2015.3689

[206] Luo Z, Tian M, Yang G, et al. Hypoxia signaling in human health and diseases: implications and prospects for therapeutics. Signal Transduct Target Ther. 2022;7(1):218. 10.1038/s41392-022-01080-1. 35798726 10.1038/s41392-022-01080-1 PMC9261907

[207] Duan C. Hypoxia-inducible factor 3 biology: complexities and emerging themes. Am J Physiol Cell Physiol. 2016;310(4):C260–9. 10.1152/ajpcell.00315.2015. 26561641 10.1152/ajpcell.00315.2015

[208] Zhang P, Yao Q, Lu L, et al. Hypoxia-inducible factor 3 is an oxygen-dependent transcription activator and regulates a distinct transcriptional response to hypoxia. Cell Rep. 2014;6(6):1110–21. 10.1016/j.celrep.2014.02.011. 24613356 10.1016/j.celrep.2014.02.011

[209] Reichetzeder C. Overweight and obesity in pregnancy: their impact on epigenetics. Eur J Clin Nutr. 2021;75(12):1710–22. 10.1038/s41430-021-00905-6. 34230629 10.1038/s41430-021-00905-6 PMC8636269

[210] Leddy MA, Power ML, Schulkin J. The impact of maternal obesity on maternal and fetal health. Rev Obstet Gynecol. 2008;1(4):170–8. 19173021 PMC2621047

[211] Persson M, Johansson S, Villamor E, et al. Maternal overweight and obesity and risks of severe birth-asphyxia-related complications in term infants: a population-based cohort study in Sweden. PLoS Med. 2014;11(5):e1001648. 10.1371/journal.pmed.1001648. 24845218 10.1371/journal.pmed.1001648 PMC4028185

[212] Godfrey KM, Reynolds RM, Prescott SL, et al. Influence of maternal obesity on the long-term health of offspring. Lancet Diabetes Endocrinol. 2017;5(1):53–64. 10.1016/s2213-8587(16)30107-3. 27743978 10.1016/S2213-8587(16)30107-3 PMC5245733

[213] Ma Z, Wang Y, Quan Y, et al. Maternal obesity alters methylation level of cytosine in CpG island for epigenetic inheritance in fetal umbilical cord blood. Hum Genomics. 2022;16(1):34. 10.1186/s40246-022-00410-2. 36045397 10.1186/s40246-022-00410-2 PMC9429776

[214] Zohourian N, Brown JA. Current trends in clinical trials and the development of small molecule epigenetic inhibitors as cancer therapeutics. Epigenomics. 2024;16(9):671–80. 10.2217/epi-2023-0443. 38639711 10.2217/epi-2023-0443 PMC11233149

[215] Song P, Li B. New generation of clinical epigenetics analysis and diagnosis for precision medicine. Diagnostics (Basel). 2025;15(12):1539. 10.3390/diagnostics15121539. 40564859 10.3390/diagnostics15121539 PMC12192280

[216] Tetorou K, Sisa C, Iqbal A, et al. Current therapies for neonatal hypoxic-ischaemic and infection-sensitised hypoxic-ischaemic brain damage. Front Synaptic Neurosci. 2021;13:709301. 10.3389/fnsyn.2021.709301. 34504417 10.3389/fnsyn.2021.709301 PMC8421799

[217] Jaworska J, Zalewska T, Sypecka J, et al. Effect of the HDAC inhibitor, sodium butyrate, on neurogenesis in a rat model of neonatal hypoxia-ischemia: potential mechanism of action. Mol Neurobiol. 2019;56(9):6341–70. 10.1007/s12035-019-1518-1. 30767185 10.1007/s12035-019-1518-1 PMC6682584

